# Mitochondrial Protein Synthesis Is Essential for Terminal Differentiation of CD45^–^ TER119^–^Erythroid and Lymphoid Progenitors

**DOI:** 10.1016/j.isci.2020.101654

**Published:** 2020-10-07

**Authors:** Kazuhito Gotoh, Yuya Kunisaki, Soichi Mizuguchi, Daiki Setoyama, Kentaro Hosokawa, Hisayuki Yao, Yuya Nakashima, Mikako Yagi, Takeshi Uchiumi, Yuichiro Semba, Jumpei Nogami, Koichi Akashi, Fumio Arai, Dongchon Kang

**Affiliations:** 1 Department of Clinical Chemistry and Laboratory Medicine, Graduate School of Medical Sciences, Kyushu University, Fukuoka 812-8582, Japan; 2 Department of Stem Cell Biology and Medicine/Cancer Stem Cell Research, Graduate School of Medical Sciences, Kyushu University, Fukuoka 812-8582, Japan; 3 Department of Medicine and Biosystemic Sciences, Graduate School of Medical Sciences, Kyushu University, Fukuoka 812-8582, Japan; 4 Center for Cellular and Molecular Medicine, Kyushu University Hospital, Fukuoka 812-8582, Japan; 5 Department of Health Sciences, Graduate School of Medical Sciences, Kyushu University, Fukuoka 812-8582, Japan

**Keywords:** Developmental Genetics, Molecular Biology

## Abstract

p32/C1qbp regulates mitochondrial protein synthesis and is essential for oxidative phosphorylation in mitochondria. Although dysfunction of p32/C1qbp impairs fetal development and immune responses, its role in hematopoietic differentiation remains unclear. Here, we found that mitochondrial dysfunction affected terminal differentiation of newly identified erythroid/B-lymphoid progenitors among CD45^–^ Ter119^–^ CD31^–^ triple-negative cells (TNCs) in bone marrow. Hematopoietic cell-specific genetic deletion of p32/C1qbp (p32cKO) in mice caused anemia and B-lymphopenia without reduction of hematopoietic stem/progenitor cells. In addition, p32cKO mice were susceptible to hematopoietic stress with delayed recovery from anemia. p32/C1qbp-deficient CD51^–^ TNCs exhibited impaired mitochondrial oxidation that consequently led to inactivation of mTORC1 signaling, which is essential for erythropoiesis. These findings uncover the importance of mitochondria, especially at the stage of TNCs during erythropoiesis, suggesting that dysregulation of mitochondrial protein synthesis is a cause of anemia and B-lymphopenia with an unknown pathology.

## Highlights

• p32/C1qbp is essential for development of erythrocytes and B-lymphocytes
• p32/C1qbp is necessary for terminal erythrocyte differentiation from CD44^+^ CD51^–^ TNCs
• p32/C1qbp regulates mitochondrial OXPHOS and mTORC1 signaling pathway in CD51^–^ TNCs

## Graphical Abstract

**Figure:**
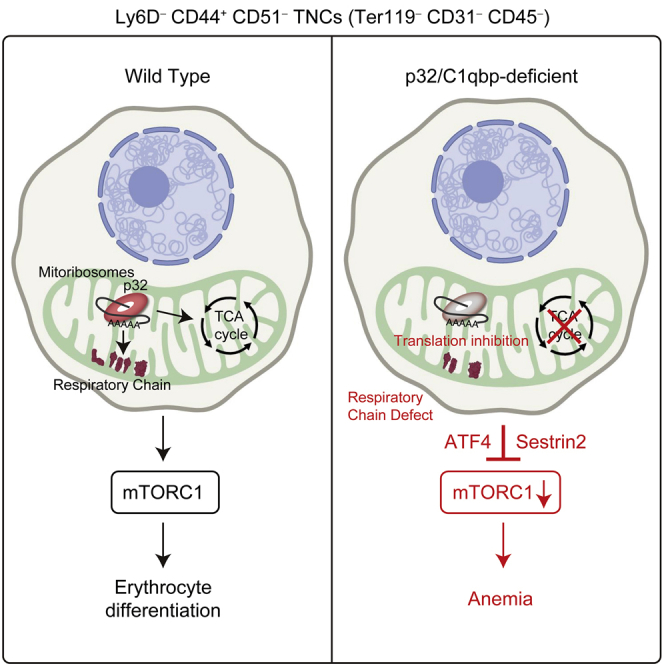

## Introduction

Mitochondria are cellular organelles involved in multiple cellular functions such as oxidative phosphorylation (OXPHOS), energy metabolism, production of reactive oxygen species, iron homeostasis, signal transduction, and apoptosis (Nunnari and Suomalainen, 2012; Spinelli and Haigis, 2018; Tait and Green, 2012). Mitochondria have their own unique transcriptional system in which mitochondrial DNA (mtDNA) encoding rRNA, tRNA, and proteins comprising the respiratory chain is transcribed in response to cellular dynamics, including mitochondrial replication, which is regulated by mitochondrial transcription factor A (TFAM) (Kang et al., 2007; Uchiumi and Kang, 2017).

p32, also known as complement component 1, q subcomponent-binding protein (p32/C1qbp), is a multifunctional chaperone protein associated with TFAM mainly localized in the mitochondrial matrix. p32/C1qbp interacts with mitochondrial mRNA and is required for mitochondrial ribosome (mitoribosome) formation to synthesize proteins within mitochondria (Leucci et al., 2016; Muta et al., 1997; Petersen-Mahrt et al., 1999; Yagi et al., 2012). Dysfunction of p32/C1qbp impairs fetal development and immune responses, and its genetic mutations are related to human diseases such as cardiomyopathy and progressive external ophthalmoplegia (Feichtinger et al., 2017; Gotoh et al., 2018).

Since initial studies reported that mitochondrial translation inhibitors (e.g. chloramphenicol [CAM]) induce bone marrow suppression and anemia (Nagao and Mauer, 1969; Yunis et al., 1970), mitochondrial dysfunction has been implicated in various hematopoietic disorders such as bone marrow failure syndromes (Cappelli et al., 2013; Kim et al., 2015). In addition, recent evidence supports a critical role of mitochondria clearance (mitophagy) in self-renewal of hematopoietic stem cells (HSCs) (Anso et al., 2017; Ito et al., 2012, 2016; Luchsinger et al., 2016).

Hematopoiesis is a multistep process originating from HSCs at the top of the hematopoietic hierarchy, which is finely regulated by cell-intrinsic transcription factors, extrinsic cytokines, and metabolic controls (Asada et al., 2017; Ito and Suda, 2014; Kunisaki et al., 2013; Lu et al., 2019; Wei and Frenette, 2018). HSCs are located in bone marrow where they differentiate into all blood lineages. In bone marrow, non-hematopoietic stromal cell populations exist as constituents of hematopoietic microenvironments supporting HSC maintenance and differentiation (Mendez-Ferrer et al., 2010; Pinho and Frenette, 2019; Pinho et al., 2013; Sacchetti et al., 2007). Although the non-hematopoietic cell fraction of bone marrow was classically isolated as CD45^–^ Ter119^–^ CD31^–^ cells (hereafter referred to as triple-negative cells; TNCs), erythroid and lymphoid progenitors, which rapidly expand to replenish progenies in cases of hemolytic crises, have been newly identified among CD51^–^ TNCs (Boulais et al., 2018). Cell differentiation is a dynamic process during which numerous transcriptional and metabolic changes occur to assign the progenies with specific functions and characteristics. With recent advances in genomic technologies, nuclear transcription factors essential for lineage commitments have been identified. However, the roles of mitochondrial protein synthesis over the course of differentiation are yet to be clarified.

To address this issue, we generated hematopoietic-specific p32/C1qbp-deficient mice, in which mitochondria were found to be structurally and functionally impaired, and investigated the relationships between dysregulation of mitochondrial protein synthesis and hematopoietic differentiation in a steady-state and under hematopoietic stress.

## Results

### p32/C1qbp Is Essential for Development of Erythrocytes and B-Lymphocytes

We previously reported that p32/C1qbp-deficient mice are embryonic lethal owing to loss of mitochondrial translation (Yagi et al., 2012). To investigate the functions of p32/C1qbp in hematopoietic cells, we generated a hematopoietic-specific p32/C1qbp conditional knockout (p32cKO) mouse strain by crossing p32/C1qbp^flox/flox^ mice with Vav1-Cre transgenic mice. Protein expression analyses of bone marrow cells isolated from p32cKO mice (p32^flox/flox^ Vav1-Cre^+^) and control littermates (p32^flox/flox^ Vav1-Cre^–^) confirmed that p32/C1qbp protein was deleted efficiently in the bone marrow cells of p32cKO mice (Figure S1A). Coincident with a previous report showing that mitochondrial p32/C1qbp is required for maturation of mitochondrial rRNA and synthesis of mitochondria-encoded proteins (Leucci et al., 2016; Yagi et al., 2012), we found that 16S rRNA levels in the bone marrow cells was reduced significantly by the loss of p32/C1qbp (Figures S1B and S1C). Furthermore, we examined the expression levels of respiratory chain proteins by immunoblotting. The protein levels of complex I and IV, which include mtDNA-encoded subunits, were decreased significantly in p32/C1qbp-deficient (p32^−/−^) bone marrow cells (Figure S1D). These results indicated that synthesis of proteins associated with the mitochondrial respiratory chain was dependent on p32/C1qbp in hematopoietic cells, which prompted us to investigate hematopoiesis in p32cKO mice.

**Figure 1:**
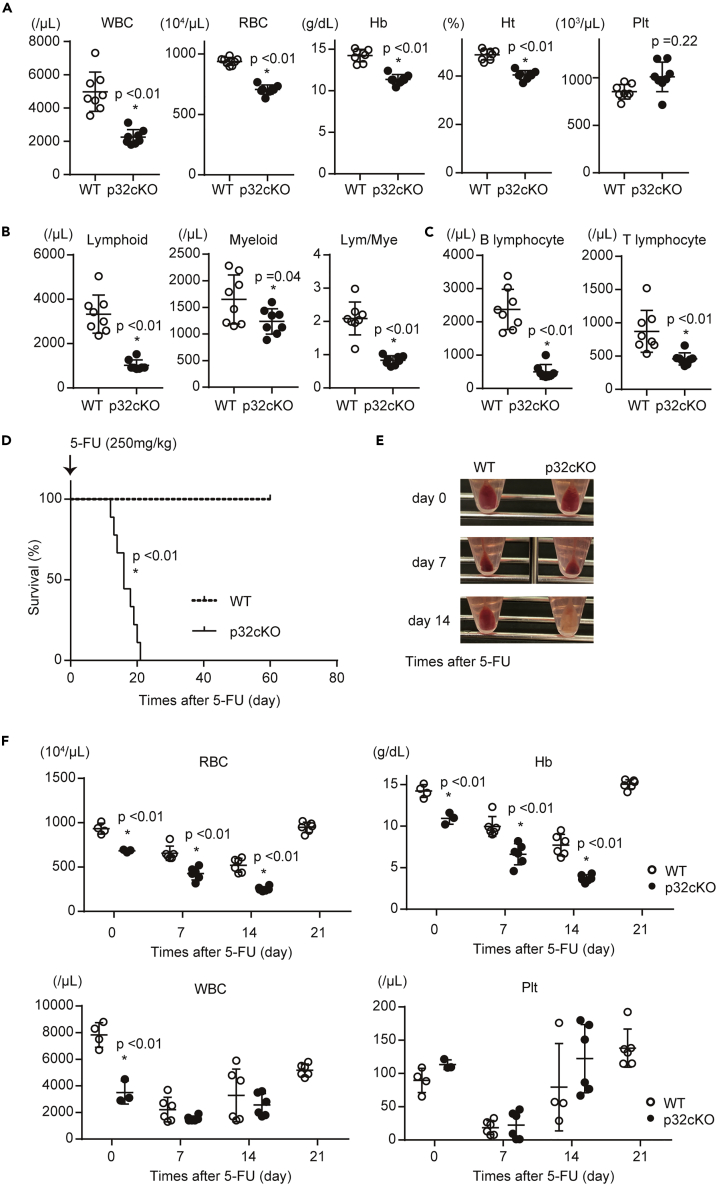
p32/C1qbp is Essential for Development of Erythrocytes and B-Lymphocytes (A) WBCs, RBCs, hemoglobin concentration (Hb), hematocrit (Ht), and the platelet (Plt) count in peripheral blood from 8-12-week-old WT (open circle, n = 8) and p32cKO (closed squares, n = 8) mice. (B and C) Numbers of lymphoid cells (Gr-1^–^ CD11b^–^), myeloid cells (Gr-1^+^ CD11b^+^), B-lymphocytes (CD19^+^ CD3^–^ Gr-1^–^ CD11b^–^), and T-lymphocytes (CD19^–^ CD3^+^ Gr-1^–^ CD11b^–^) in the peripheral blood. (D) Kaplan-Meier plots of age-matched WT and p32cKO mice (n = 9) treated with 5-FU (250 mg/kg). (E) Appearance of bone marrow pellets from WT and p32cKO mice after 5-FU injection. (F) WBCs, RBCs, Hb, and Plts in peripheral blood from WT (open circle, n = 4–6) and p32cKO (closed squares, n = 4–6) mice after 5-FU injection (250 mg/kg). In (A–C) and (F), data are shown as means ± SD. ∗p < 0.05 versus WT mice. Data are representative at least three (A–F) independent experiments. See also Figures S1 and S2.

We thus examined relationships between p32/C1qbp and hematopoiesis. p32cKO mice at the age of 8–12 weeks old displayed anemia and a decline of white blood cell (WBC) counts in the peripheral blood (Figure 1A). Among the WBCs, the number of both B- and T-lymphocytes was prominently affected more than those of myeloid cells (Figures 1B, 1C and S2A). To clarify what stages of hematopoietic precursors were functionally impaired, we performed bone marrow reconstitution analyses, in which total bone marrow cells from WT or p32cKO mice were transplanted into lethally irradiated WT recipients. Although anemia and reduction of white blood cells and B-lymphocytes were observed by 4 weeks after bone marrow transplantation with p32cKO BM cells, which sustained until at least 12 weeks, the numbers of T-lymphocytes and myeloid cells were gradually reduced in mice transplanted from p32cKO mice (Figures S2B and S2C). These findings suggest that differentiation of erythroid and lymphoid committed progenitors was effected more prominently rather than hematopoietic stem/progenitor cells.

Next, we examined the role of p32/C1qbp in hematopoietic recovery after administration of a cytotoxic drug, 5-fluorouracil (5-FU). After a single injection of 5-FU (250 mg/kg), all control mice survived, even though they exhibited transient pancytopenia with recovery by day 21 (Figures 1D–1F). In contrast, p32cKO mice were moribund within 21 days due to delayed recovery from bone marrow suppression including severe anemia (Figures 1D–1F). The bleached color of whole bone marrow cells isolated from p32cKO mice corroborated the severity of anemia (Figure 1E). Similar results were obtained after weekly 5-FU administrations (150 mg/kg) (Figure S2D). These results indicate that p32/C1qbp is essential for development of erythroid and B-lymphoid pools in the periphery and their replenishment under hematopoietic suppressing stresses.

### p32/C1qbp Deficiency Prohibits Terminal Differentiation of Erythrocytes and B-Lymphocytes

**Figure 2:**
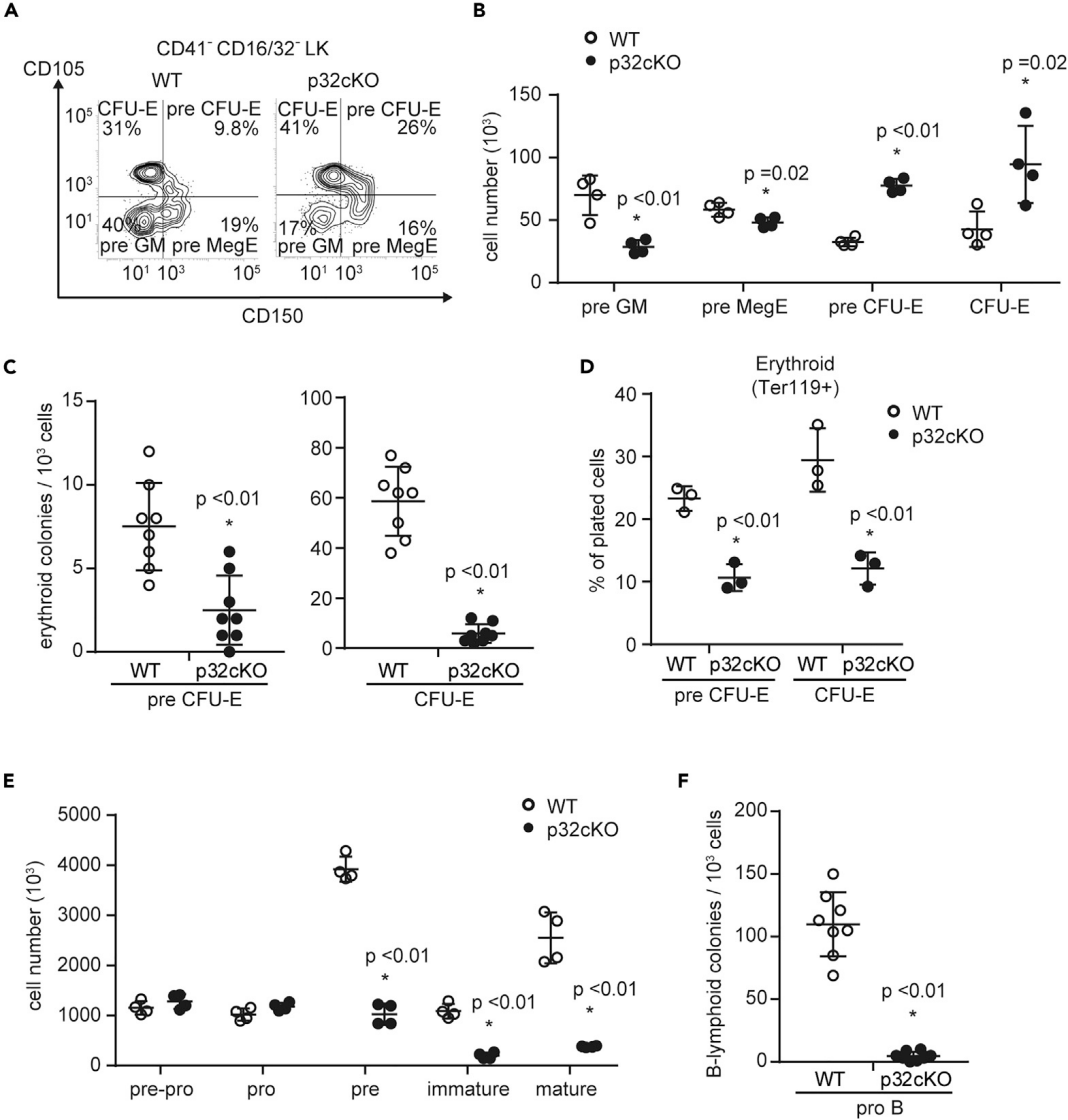
p32/C1qbp Deficiency Prohibits Terminal Differentiation of Erythrocytes and B-Lymphocytes (A) Representative flow cytometry plots of pre-GMs, pre-MegEs, pre-CFU-E cells, and CFU-E cells in bone marrow (BM) of WT and p32cKO mice. (B and E) Numbers of pre-GMs (CD150 ^–^ CD105^–^ CD41^–^ CD16/32^–^ LK), pre-MegEs (CD150^+^ CD105^–^ CD41^–^ CD16/32^–^ LK), preCFU-Es (CD150^+^ CD105^+^ CD41^–^ CD16/32^–^ LK), CFU-Es (CD150^–^ CD105^+^ CD41^–^ CD16/32^–^ LK) (B) and pre-pro- (B220^+^ CD19^–^ CD43^+^ IgM^–^), pro- (B220^+^ CD19^+^ CD43^+^ IgM^–^), pre- (B220^+^ CD19^+^ CD43^–^ IgM^–^), immature (B220^+^ CD19^+^ CD43^–^ IgM^+^), and mature (B220^++^ CD19^+^ CD43^–^ IgM^+–^) B cells (E) in BM from WT and p32cKO mice. (C and F) Sorted pre-CFU-Es, CFU-Es (C), and pro-B cells (F) from WT and p32cKO mice were plated at low densities (1000 cells/well) in methylcellulose (Stem Cell Technologies, Cat#: M3334 [CFU-E] Cat#: 3630 [CFU pre-B + 25ng/mL SCF]). Erythroid and B-lymphoid colonies were enumerated at day 2 (C) and day 5 (F). (D) Sorted pre-CFU-E and CFU-E cells from WT and p32cKO mice were seeded in liquid culture with cytokines (stem cell factor, IL-3, IL-6, erythropoietin, and thrombopoietin) for 48 hr. The erythroid (Ter119^+^) lineage potential of sorted preCFU-E and CFU-E cells is shown. Cell numbers in each population were normalized as the percentage of total cells plated per well (% of cells plated). (B–F) Data are shown as means ± SD. ∗p < 0.05 versus WT mice. Data are representative at least three (A–F) independent experiments. See also Figures S2 and S3.

We next examined the bone marrow cells in p32cKO mice and found no significant changes in the numbers of HSCs and multipotent, common myeloid, or common lymphoid progenitors (Figures S2E–S2G). To determine which stage of differentiation was affected, we analyzed lineage-committed progenitors in the bone marrow. Multipotent progenitor cells 2 generates pre-megakaryocyte-erythrocytes (pre-MegEs) (Pietras et al., 2015). Pre-MegEs are bipotent cells that are upstream of more committed erythroid-restricted progenitor (pre-CFU-E) and colony-forming unit erythroid (CFU-E) cells. The numbers and proportions of pre-CFU-E and CFU-E cells were increased significantly in the bone marrow of p32cKO mice (Figures 2A and 2B, and S3A). We sorted pre-CFU-E and CFU-E cells from p32cKO bone marrow and examined their capacities for differentiation into erythrocytes *in vitro*. As a result, p32/C1qbp deficiency impaired the differentiation of pre-CFU-E and CFU-E cells into erythroid cells *in vitro* (Figures 2C and 2D), suggesting that p32/C1qbp is important for erythroid differentiation after the CFU-E cell stage. Development of B-lymphocytes in bone marrow progresses following the order of pre-pro-B, pro-B, pre-B, immature B, and mature B cells (Nagasawa, 2006). The numbers of B-lymphocytes at the stage later than pre-B cells were reduced significantly in the bone marrow of p32cKO mice compared to those of controls (Figures 2E and S3B). To assess the differentiation capacities of pro-B cells, we sorted pro-B cells and evaluated their ability to form colonies of pre-B cells in culture. As observed in the erythroid lineage, the loss of p32/C1qbp impaired a colony-forming activity of B-lymphoid cells (Figure 2F). Taken together, these results indicate that terminal differentiation of erythrocytes and B-lymphocytes is disrupted by p32/C1qbp deficiency.

### Mitoribosomes Are Essential for CD45^–^ Erythroid and B-Lymphoid Progenitor Differentiation

**Figure 3:**
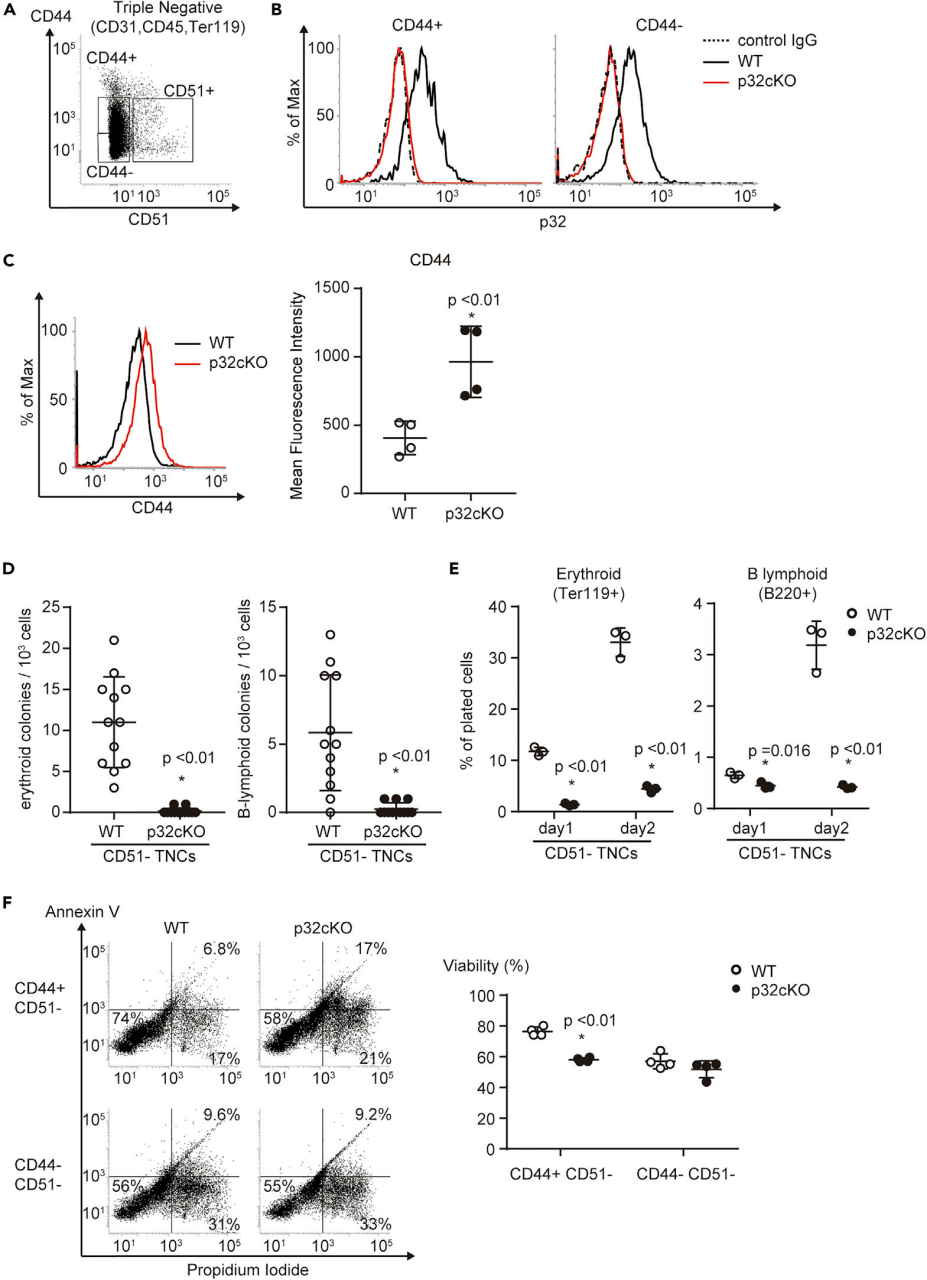
Mitoribosomes are Essential for CD45^–^ Erythroid and B-Lymphoid Progenitor Differentiation (A and B) Representative flow cytometry plots of CD44 ^+^ CD51^–^ and CD44^–^ CD51^–^ cells among CD31/CD45/Ter119 triple-negative cells (TNCs) in enzymatically digested bone marrow. Histograms of p32 (B) expression in CD44^+^ CD51^–^ and CD44^–^ CD51^–^cells among TNCs. The IgG isotype control is shown as a dotted line. (C) Histograms of CD44 expression in CD51^–^ TNCs. Results are shown as the mean fluorescence intensity ±SD. (D) Sorted CD51^–^ TNCs from WT and p32cKO mice were plated at low densities (1000 cells/well) in methylcellulose (Stem Cell Technologies, Cat#: M3334 [CFU-E] Cat#: 3630 [CFU pre-B + 25ng/mL SCF]). Erythroid and B-lymphoid colonies were enumerated at day 2 and day 5. (E) Quantification of Ter119^+^ (erythroid) and B220^+^ (B-lymphoid) cells that were differentiated from sorted CD51^–^ TNCs of WT and p32cKO mice seeded in liquid culture with cytokines (stem cell factor, IL-3, IL-6, erythropoietin, and thrombopoietin) under normoxia for 48 hr. Cell numbers in each population were normalized as the percentage of total cells plated per well (% of cells plated). (F) FACS analysis of cell death in CD44^+^ CD51^–^ TNCs, and CD44^–^ CD51^–^ TNCs (left). The rates of the population of Annexin V^–^/Propidium Iodide^–^ are indicated (right). In (C–F) data are shown as means ± SD. ∗p < 0.05 versus WT mice. Data are representative at least three (A–F) independent experiments. See also Figures S4.

CD45^–^ Ter119^–^ CD31^–^ TNCs were classically defined as the non-hematopoietic stromal fraction isolated from bone marrow (Mizoguchi et al., 2014; Park et al., 2012; Pinho et al., 2013). Recent studies revealed that the majority of TNCs have a hematopoietic rather than mesenchymal origin and that a CD51^–^ CD44^+^ population among TNCs exhibits erythroid and B-lymphoid progenitor signature (Boulais et al., 2018). Therefore, we hypothesized that p32/C1qbp deficiency had a prominent effect on CD51^–^ CD44^+^ triple-negative common erythroid and B-lymphoid progenitors. Flow cytometric analyses confirmed p32/C1qbp protein expression in CD51^–^ TNCs regardless of CD44 expression (Figures 3A and 3B). The expression level of CD44 was increased in p32-deficient CD51^–^ TNCs (Figure 3C). To analyze differentiation capabilities of p32^−/−^ CD51^–^ TNCs, we sorted CD51^–^ TNCs from control and p32^−/−^ bone marrow and performed *in vitro* differentiation assays. Sorted CD51^–^ TNCs were cultured in the presence of hematopoietic cytokines, [stem cell factor, interleukin (IL)-3, IL-6, IL-7, erythropoietin, and thrombopoietin] for 48 hr and then analyzed by flow cytometry. Consistent with a previous study (Boulais et al., 2018), CD51^–^ TNCs from control bone marrow expressed either TER119 or B220, indicating terminal differentiation to mature erythrocytes and B-lymphocytes (Figures 3D, 3E, and S4A). In contrast, p32^−/−^ CD51^–^ TNCs failed to commit to erythroid or B-lymphoid lineages and were prone to cell death (Figures 3D, 3E, S4A, and S4B). In addition, we analyzed CD44^+^ and CD44^–^ populations in CD51^–^ TNCs separately (Figures 3A and S4C) and found that in p32^−/−^ bone marrow the numbers of CD44^+^ CD51^–^ TNCs showed a ∼2-fold increase compared with controls, whereas CD44^–^ CD51^–^ TNCs exhibited a reduction in number (Figures S4C and S4D). Cell viability of CD44^+^ CD51^–^ TNCs decreased in p32^–/–^ bone marrow compared to controls (Figure 3F). While CD44^+^ CD51^–^ TNCs also expanded 14 days after 5FU treatments in p32^−/−^ bone marrow (Figure S4E), while severe anemia was observed in the peripheral blood (Figures 1D–1F), suggesting p32-deficiency blocks erythroid differentiation of CD44^+^ CD51^–^ TNCs. These results indicate the importance of p32/C1qbp for terminal differentiation of CD51^–^ triple-negative erythroid and B-lymphoid progenitors, of which a CD44^+^ population may be particularly influenced by p32-deficiency in a steady state and under hematopoietic stresses.

### p32/C1qbp Regulates Mitochondrial OXPHOS in CD51^–^ TNCs

**Figure 4:**
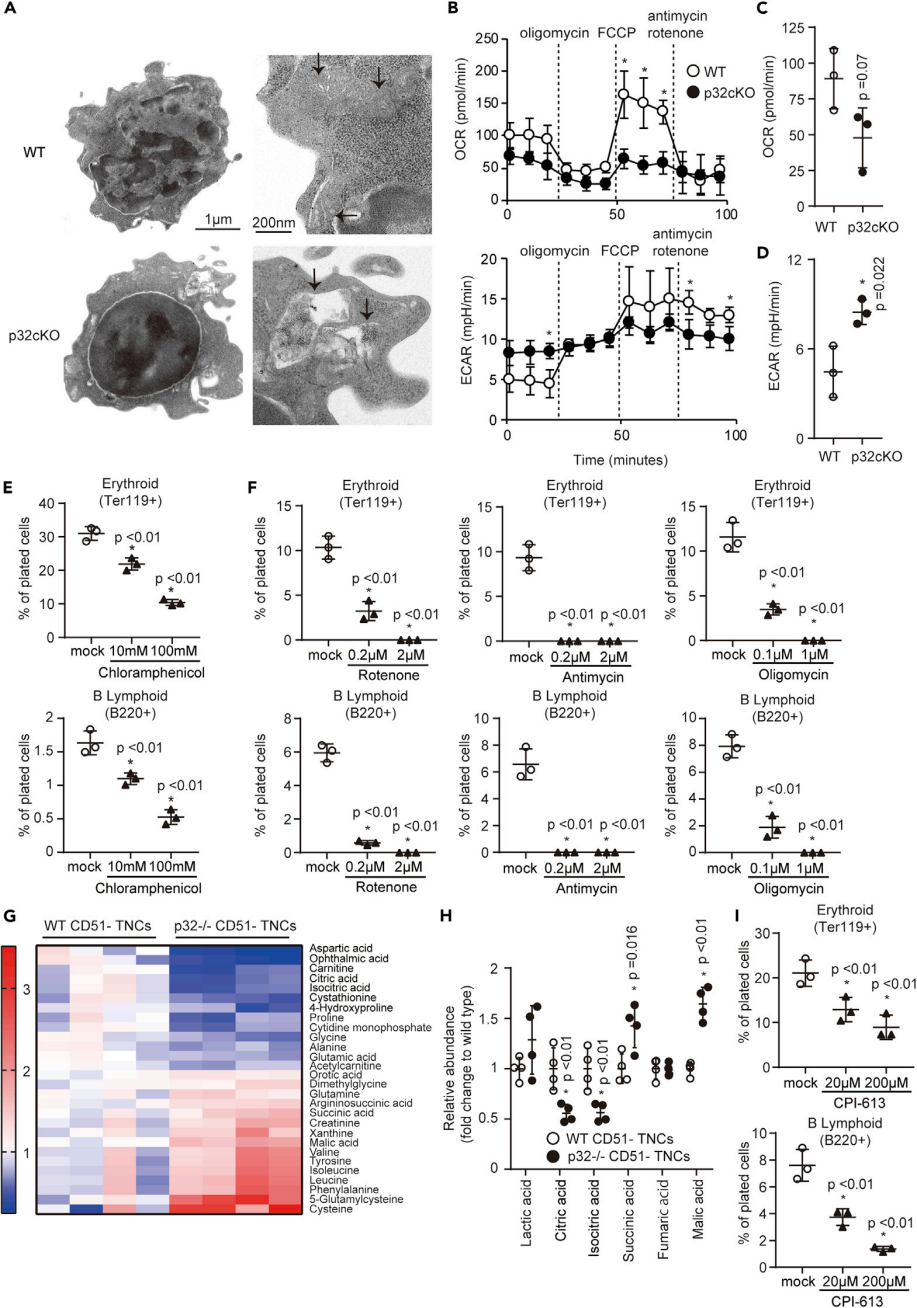
p32/C1qbp Regulates Mitochondrial OXPHOS in CD51^–^ TNCs (A) Electron microscopic images of sorted CD51^–^ TNCs. Images on the right highlight individual mitochondria (black arrows). (B–D) Measurements of the OCR and ECAR in CD51^–^ TNCs (2×10^5^ cells/well) from WT and p32cKO mice by an XF-24 extracellular flux analyzer. The real-time OCR and ECAR were determined during sequential treatments with oligomycin (ATP synthase inhibitor), FCCP, and antimycin-A/rotenone (ETC inhibitors) (B). (E, F, and I) Quantification of Ter119^+^ (erythroid) and B220^+^ (B-lymphoid) cells that were differentiated from sorted CD51^–^ TNCs of WT mice seeded in liquid culture with cytokines in the presence or absence of chloramphenicol (E), rotenone, antimycin, oligomycin (F), and CPI-613 (I) for 48 hr. Cell numbers in each population were normalized as the percentage of total cells plated per well (% of cells plated). (G) Comparisons of the amounts of metabolites between WT and p32^−/−^ CD51^–^ TNCs. Heat map of metabolites extracted from WT and p32^−/−^ CD51^–^ TNCs showing statistically significant changes (P < 0.05). (H) Comparisons of the amounts of metabolites associated with TCA cycle between WT and p32^−/−^ CD51^–^ TNCs. In B–F, H, I, data are shown as means ± SD. ∗p < 0.05 versus WT mice or DMSO controls. Data are representative at least three (A-I) independent experiments. See also Figures S5.

To explore the mechanisms by which p32/C1qbp was involved in the differentiation process of CD51^–^ TNCs into erythrocytes and B-lymphocytes, we analyzed structure and functions of mitochondria in CD51^–^ TNCs. Electron microscopy revealed the abnormal morphologies of mitochondria as impaired cristae organization and the appearance of abnormal components in p32^−/−^ CD51^–^ TNCs (Figures 4A and S5A), although there was no difference in the mitochondrial mass or membrane potential measured by flow cytometry with MitoTracker Green FM and tetramethylrhodamine methyl ester, respectively (Figures S5B and S5C). Using an XF-24 extracellular flux analyzer, we next measured the oxygen consumption rate (OCR) as an indicator of mitochondrial OXPHOS and the extracellular acidification rate (ECAR) as an index of lactate production and glycolysis in CD51^–^ TNCs. As a result, p32^−/−^ CD51^–^ TNCs exhibited a lower OCR and higher ECAR compared with controls (Figures 4B and 4C). These results suggest that p32/C1qbp deletion impairs mitochondrial OXPHOS that promotes a metabolic shift between two major metabolic systems, OXPHOS and glycolysis, to generate ATP in CD51^–^ TNCs, as observed previously in dendritic cells (Gotoh et al., 2018). In addition, an inhibitor of mitochondrial translation, CAM, exerted inhibitory effects on erythroid and B-lymphoid differentiation of CD51^–^ TNCs, and rotenone and antimycin-A, inhibitors of the mitochondrial electron transport chain, and oligomycin, an inhibitor of mitochondrial ATP synthase, exerted the same effects on CD51^–^ TNC differentiation (Figures 4E and 4F) (Sasaki et al., 2017). To directly measure metabolites associated with mitochondrial OXPHOS, we performed a mass spectrometric analysis of sorted WT and p32^−/−^ CD51^–^ TNCs. Using a statistical cutoff (p < 0.05), we identified several metabolites that showed differential abundance in p32^−/−^ CD51^–^ TNCs (Figures 4G and S5D). Consistent with a previous study (Gotoh et al., 2018), intermediate metabolites of the tricarboxylic acid cycle, including citrate and isocitrate, were decreased in p32^−/−^ CD51^–^ TNCs (Figure 4H). We also found that pyruvate dehydrogenase (PDH) activity was decreased in p32^−/−^ CD51^–^ TNCs (Figure S5E), suggesting that p32/C1qbp regulates mitochondrial OXPHOS via PDH activity in CD51^–^ TNCs. Consistent with these results, CPI-613 (6,8-bis octanoic acid), which is a selective inhibitor of PDH and a-ketoglutarate dehydrogenase (Zachar et al., 2011), also exerted inhibitory effects on erythroid and B-lymphoid differentiation of CD51^–^ TNCs (Figure 4I).

Mitochondria are critical for heme and iron metabolism because inhibition of mitochondrial translation and OXPHOS are associated with sideroblastic anemia (Ducamp and Fleming, 2019; Fleming, 2011). Heme synthesis begins in mitochondria in which decarboxylative condensation of glycine and succinyl-coenzyme A (CoA) produces 5-aminolevulinic acid (5-ALA) that is a critical product for the porphyrin synthetic pathway. We found no reduction in the amounts of 5-ALA in p32^−/−^ CD51^–^ TNCs, although the level of glycine was decreased (Figures S5D and S5F). We also evaluated expression levels of genes associated with erythroid differentiation by qPCR and p32^−/−^ CD51^–^ TNCs exhibited no reduction in these gene expression (Figure S5G). These results suggest that p32/C1qbp plays important roles in terminal differentiation of CD51^–^ triple-negative erythroid and B-lymphoid progenitors by regulating mitochondrial OXPHOS rather than heme synthesis or gene transcription.

### Gene Expression Analysis Reveals Pathways Mediated by p32/C1qbp in CD51^–^ TNCs

**Figure 5:**
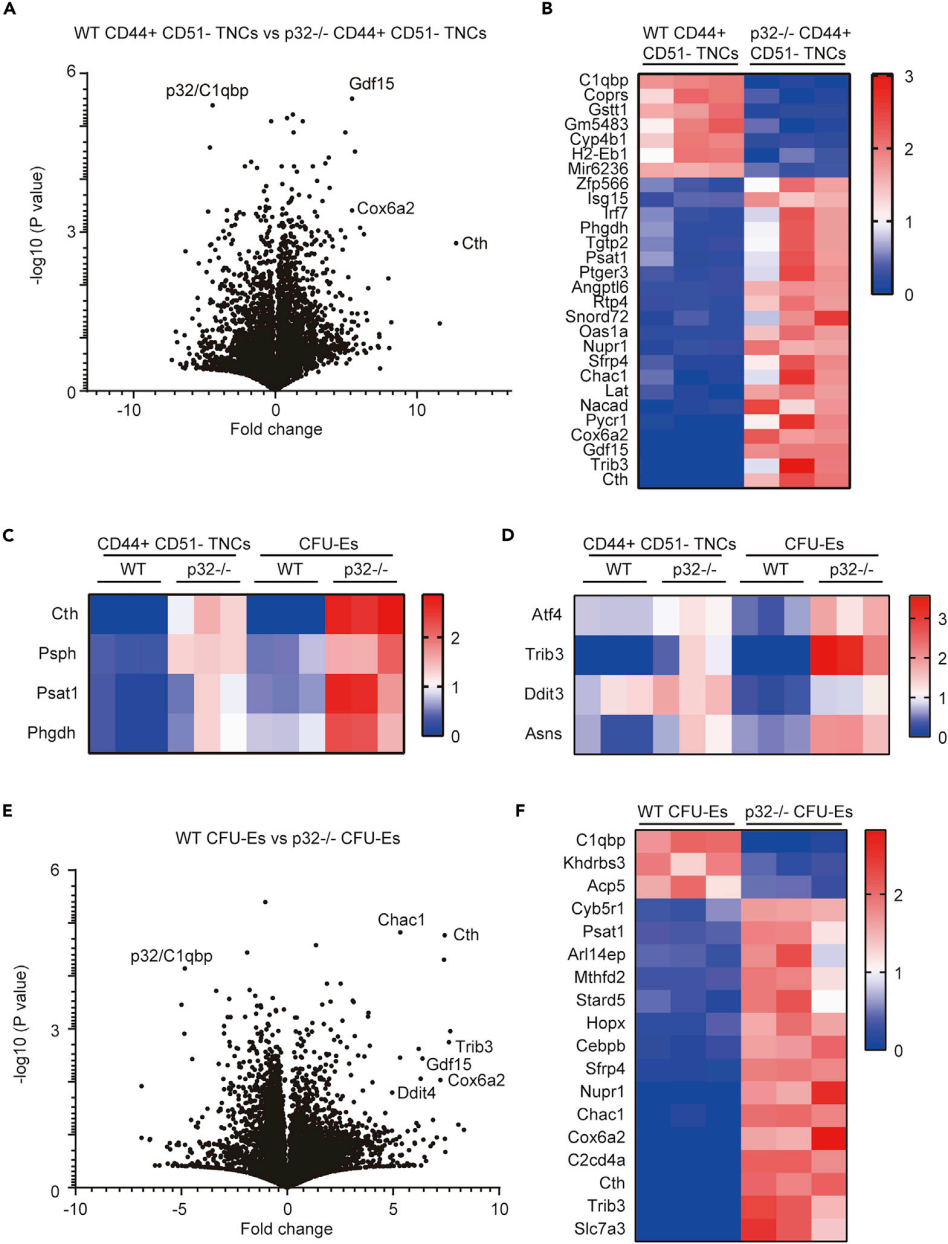
Loss of p32/C1qbp Induces Mitochondrial Integrated Stress Response in CD44^+^ CD51^–^ TNCs (A and B) Comparisons of the amounts of mRNA between WT and p32^−/−^ CD44^+^ CD51^–^ TNCs. Volcano plot (A) showing differential gene expression in CD44^+^ CD51^–^ TNCs isolated from WT (n = 3) and p32cKO (n = 3) mice. Fold change is calculated as log2(expression in p32cKO/expression in WT). Heatmap (B) of CD44^+^ CD51^–^ TNCs signature genes that are differentially expressed (adjusted P < 0.05, fold change >4) in WT (n = 3) versus p32cKO (n = 3) mice. (C and D) Heat maps of relative mRNA of the genes of amino acid metabolic pathways (C) and mitochondrial integrated stress response (D) in CD44^+^ CD51^–^ TNCs and CFU-Es isolated from WT (n = 3) and p32cKO (n = 3). (E and F) Comparisons of the amounts of mRNA between WT and p32^−/−^ CFU-Es. Volcano plot (E) showing differential gene expression in CFU-Es isolated from WT (n = 3) and p32cKO (n = 3) mice. Fold change is calculated as log2(expression in p32cKO/expression in WT). Heatmap (F) of CFU-Es signature genes that are differentially expressed (adjusted P < 0.05, fold change >4) in WT (n = 3) versus p32cKO (n = 3) mice. CD44^+^ CD51^–^ TNCs and CFU-Es from WT (n = 3) and p32cKO (n = 3) mice were isolated on different days. Further processing and sequencing was performed with all twelve samples simultaneously. See also Figures S6.

To explore further mechanisms by which p32/C1qbp was involved in the differentiation process of CD51^–^ TNCs into erythrocytes and B-lymphocytes, we analyzed differentially expressed genes in sorted CFU-E cells and CD44^+^ CD51^–^ TNCs, which were enriched with more erythroid progenitors than the CD44^–^ CD51^–^ fraction, from control and p32cKO bone marrow (Figure 5A). Consistent with a previous study, the gene expression involved in erythroid differentiation of CD44^+^ CD51^–^ TNCs was comparable with that of CFU-Es (Figure S6A). Previous studies show that inhibition of mitochondrial translation induces alterations in amino acid metabolism pathways and mitochondrial integrated stress responses (mtISRs) (Bao et al., 2016) (Quiros et al., 2017) (Forsstrom et al., 2019). RNA sequencing analyses revealed that expression of several genes involved in amino acid metabolism (Cth, Psph, Psat1 and Phgdh) increased in p32^−/−^ CD44^+^ CD51^-^ TNCs compared to WT (Figures 5B, 5C, and S6B). In addition, ATF4-dependent mtISR-related genes (Trib3, Chac1, Ddit3 and Asns) were upregulated in p32^−/−^ CD44^+^ CD51^–^ TNCs (Figures 5B, 5D, and S6C). Similar results were obtained with CFU-E cells (Figures 5C–5F), suggesting that p32/C1qbp-mediated pathways in erythroid differentiation are shared by CD44^+^ CD51^–^ TNCs and CFU-E cells.

### p32/C1qbp Regulates the mTORC1 Signaling Pathway in CD51^–^ TNCs

**Figure 6:**
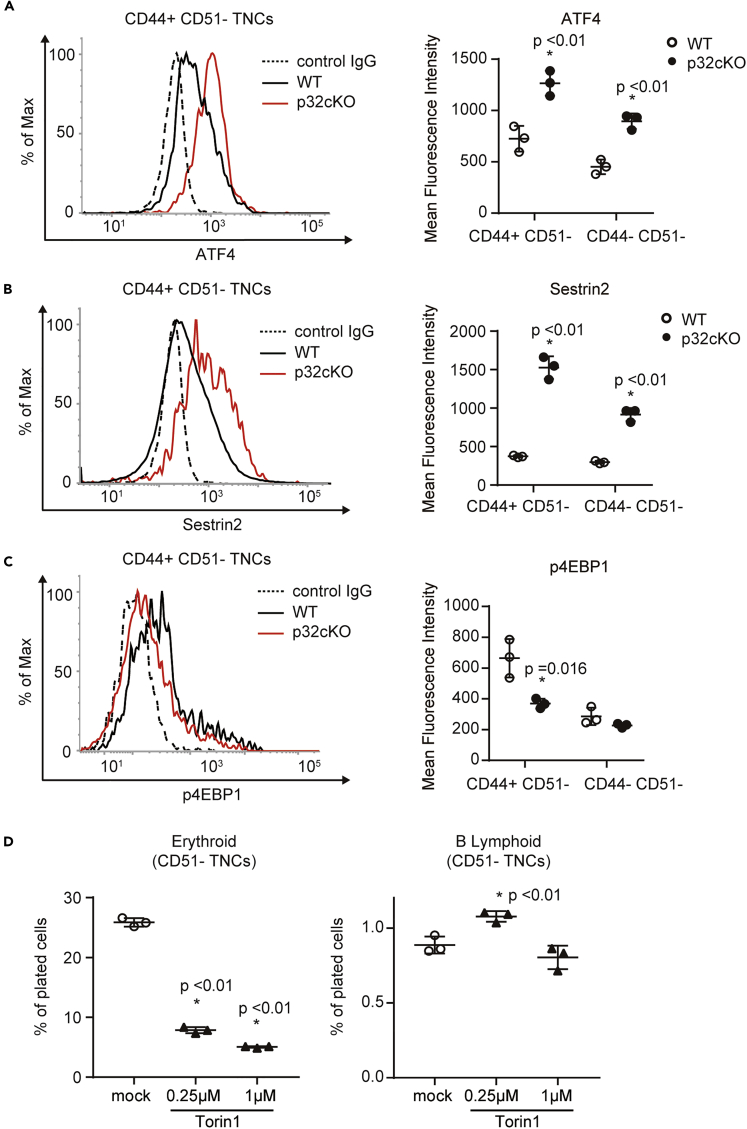
p32/C1qbp Regulates the mTORC1 Signaling Pathway in CD51^–^ TNCs (A–C) Flow cytometry histograms and quantification of the expression of ATF4 (A), Sestrin2 (B), and p4EBP1 (C) in CD44 ^+^ CD51^–^ TNCs from WT (n = 3) and p32cKO (n = 3) mice. IgG isotype controls are depicted as dotted lines. Results are shown as the mean fluorescence intensity ±SD. (D) Quantification of Ter119^+^ (erythroid) and B220^+^ (B-lymphoid) cells that were differentiated from sorted CD51^–^ TNCs of WT mice seeded in liquid culture with cytokines in the presence or absence of Torin 1 for 48 hr. Cell numbers in each population were normalized as the percentage of total cells plated per well (% of cells plated). In A–D, data are shown as means ± SD. ∗p < 0.05 versus WT mice or DMSO controls. Data are representative of three independent experiments.

We previously reported impaired mammalian Target of Rapamycin (mTOR) signaling in p32/C1qbp-deficient cardiomyocytes (Saito et al., 2017). mTOR is a serine/threonine kinase that plays critical roles in regulation of metabolic homeostasis such as mRNA translation, lipid biosynthesis, autophagy, and mitochondrial biogenesis (Saxton and Sabatini, 2017). mTOR forms a catalytic subunit of two distinct protein complexes, known as mTOR complex (mTORC) 1 and 2. In particular, mTORC1 is a key regulator of protein translation, which supports mitochondrial biogenesis and is necessary during erythropoiesis (Knight et al., 2014) (Liu et al., 2017). To investigate whether p32/C1qbp regulated the mTORC1 signaling pathway in CD51^–^ TNCs, we analyzed protein expression of negative regulators of mTORC1, ATF4 and sestrin-2, in CD44^+^ CD51^–^ TNCs from control and p32cKO bone marrow. Expression of both ATF4 and sestrin-2 was elevated in p32^−/−^ CD51^–^ TNCs (Figures 6A and 6B). Eukaryotic translation initiation factor 4E binding protein 1 (4E-BP1) is a major effector of mTOR signaling, and phosphorylation of 4E-BP1 on amino acids Thr37 and/or Thr46 represents the activity of mTORC1 (Livingstone and Bidinosti, 2012; Saxton and Sabatini, 2017). Expression of phosphorylated 4E-BP1 (p4EBP1) measured by flow cytometry was reduced significantly in p32^−/−^ CD44^+^ CD51^–^ TNCs compared with controls (Figure 6C). In addition, Torin 1, an mTOR inhibitor, suppressed the differentiation of CD44^+^ CD51^–^ TNCs into erythrocytes but not B-lymphocytes (Figure 6D). Collectively, p32/C1qbp-mediated mTORC1 regulation is crucial for at least CD44^+^ CD51^–^ TNCs to differentiate into erythrocytes.

### p32cKO Mice Are Susceptible to Hemolysis Due to Erythroid Differentiation Failure

**Figure 7:**
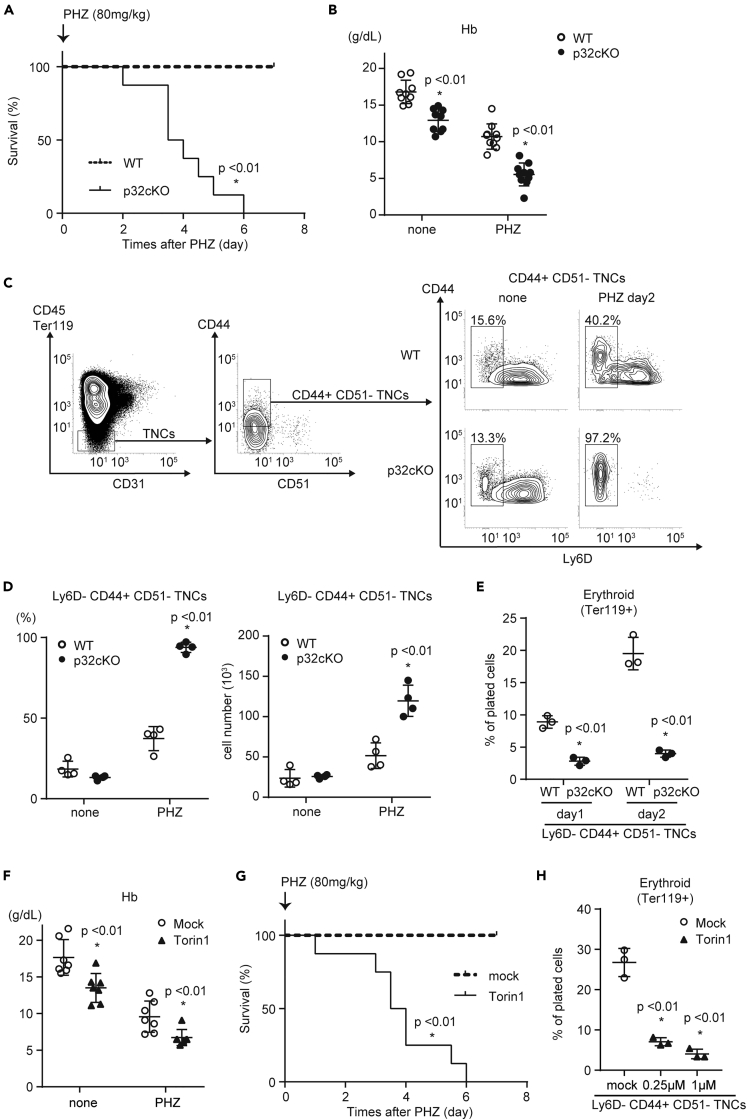
p32cKO Mice are Susceptible to Hemolysis Due to Erythroid Differentiation Failure (A) Kaplan–Meier plot of age-matched WT and p32cKO mice (n = 8 per group) treated with PHZ (80 mg/kg). (B) Hb levels in peripheral blood from WT (n = 8) and p32cKO (n = 8) mice treated with PHZ (80 mg/kg). (C and D) Representative flow cytometry plots (C), and proportions and absolute numbers (D) of Ly6D^–^ CD44^+^ CD51^–^ TNCs in enzymatically digested bone marrow from WT and p32cKO mice after PHZ injection. n = 4 mice per group. (E) Quantification of Ter119^+^ (erythroid) and B220^+^ (B-lymphoid) cells that were differentiated from sorted Ly6D^–^ CD44^+^ CD51^–^ TNCs of WT and p32cKO mice seeded in liquid culture with cytokines for 48 hr n = 3 mice per group. (F–H) Analysis of Torin 1-treated mice after PHZ injection. n = 8 mice per group. Hb levels in the peripheral blood (F) and Kaplan–Meier plot of WT mice (n = 8) treated with PHZ (80 mg/kg) after Torin 1 (20 mg/kg) injection (G). Quantification of Ter119^+^ (erythroid) and B220^+^ (B-lymphoid) cells that were differentiated from sorted Ly6D^–^ CD44^+^ CD51^–^ TNCs of WT mice (H). Cell numbers in each population were normalized as the percentage of total cells plated per well (% of cells plated). Data are shown as means ± SD. ∗p < 0.05 versus WT mice or DMSO controls. Data are representative of three independent experiments. See also Figures S7.

We further investigated whether recovery of erythrocytes could be dependent of CD44^+^ CD51^–^ TNCs using a phenylhydrazine (PHZ)-induced hemolytic anemia model. When control and p32cKO mice were treated with a single dose of PHZ (80 mg/kg), all p32cKO mice died due to severe anemia within 6 days after injection, whereas control mice showed a 100% survival rate (Figures 7A and 7B). In the bone marrow, the proportions and numbers of Ly6D^–^ CD44^+^ CD51^–^ TNCs, which were enriched with pre-proerythroblasts (Boulais et al., 2018), were increased significantly in p32cKO mice after PHZ administration (Figures 7C and 7D). Sorted p32^−/−^ Ly6D^–^ CD44^+^ CD51^–^ TNCs failed to give rise to mature erythroid colonies *in vitro* (Figure 7E). We also examined the numbers of pre-CFU-Es and CFU-Es in the bone marrow after PHZ injection (Figures S7A and S7B). The numbers of pre-CFU-Es increased and those of CFU-Es decreased in p32cKO mice compared to controls, suggesting erythroid differentiation was blocked at the stage between these two populations. These results imply that expansion of Ly6D^–^ CD44^+^ CD51^–^ TNCs contributes to the recovery from hemolytic stresses in cooperation with CFU-Es.

Our data indicate that the p32/C1qbp-mTORC1 axis is essential for terminal differentiation of CD51^–^ TNCs into erythrocytes (Figure 6). Next, we treated C57/BL6 mice with Torin 1. As observed in p32cKO mice, Torin 1-treated mice exhibited severe anemia and no mice survived after a single injection of PHZ (80 mg/kg) (Figures 7F and 7G). In addition, sorted Ly6D^–^ CD44^+^ CD51^–^ TNCs after treatment with Torin 1 failed to give rise to mature erythroid colonies *in vitro* (Figure 7H). Taken together, these data demonstrate that p32/C1qbp is important for activation of mTORC1 signaling necessary for terminal erythrocyte differentiation from CD44^+^ CD51^–^ TNCs in acute hemolytic crises.

## Discussion

Collectively, the present data show that p32/C1qbp, which has been shown to regulate mitochondrial protein synthesis (Yagi et al., 2012), is essential for terminal differentiation of CD45^–^ TER119^–^ CD31^–^ erythroid/B-lymphoid progenitors. CD45^–^ TER119^–^ CD31^–^ TNCs were classically isolated as non-hematopoietic stromal cells in bone marrow, but a recent study found that this population contains common erythroid/B-lymphoid progenitors with combinations of several surface markers such as CD51, Ly6D, and CD44 (Boulais et al., 2018; Mendez-Ferrer et al., 2010; Morikawa et al., 2009; Park et al., 2012). In particular, Ly6D^–^ CD44^+^ CD51^–^ TNCs are erythroid-committed cells able to expand and differentiate into mature erythrocytes under hemolytic stresses such as sickle cell crisis and PHZ-induced anemia in mice.

Erythropoiesis is a multistep process as a series of erythroid-committed progenitors, erythroid burst-forming unit cells, CFU-E cells, proerythroblasts, and erythroblasts, during which numerous transcriptional and metabolic changes occur (Hattangadi et al., 2011). Among them, CFU-E cells have the potential to proliferate rapidly in response to acute anemia, and the importance of mTOR signaling at the proerythroblast stage has been reported (Hattangadi et al., 2011). Our findings show that inhibition of mTOR signaling by p32/C1qbp deficiency or a selective ATP-competitive inhibitor of mTOR, Torin 1, blocked CD51^–^ TNCs including pre-proerythroblasts from differentiating into erythrocytes. Expansion of CD44^+^ CD51^–^ TNCs contributes to recovery from hemolytic crises in cooperation with the bona fide erythroid precursor, CFU-Es. Furthermore, p32/C1qbp-mediated pathways in erythroid differentiation are shared by CD44^+^ CD51^–^ TNCs and CFU-E cells.

mTORC1 is a protein complex composed of mTOR, a key regulator of protein synthesis that also supports mitochondrial functions. mTORC1-mediated protein translation is closely associated with mitochondrial biogenesis in erythropoiesis (Liu et al., 2017) (Knight et al., 2014) (Malik et al., 2019). During erythropoiesis, mTORC1 signaling is upregulated and accompanied by increases of the mitochondrial mass, mtDNA, MMP, and protein synthesis. Our electron microscopic analysis showed that CD51^–^ TNCs possessed abundant mitochondria and ribosomes unlike other hematopoietic cells (Figure 4A). These findings may explain our observations that mitochondrial dysfunctions caused by p32/C1qbp deficiency prominently influenced the differentiation of CD51^–^ TNCs, and that Torin 1 was less effective for B-lymphocyte differentiation.

Furthermore, our data suggest that p32/C1qbp regulates mTORC1 signaling by upregulating ATF4 and Sestrin2 that negatively regulate mTORC1. p32/C1qbp deficiency impairs the mitochondrial structure and OXPHOS in CD51^–^ TNCs, which may be involved in the molecular pathway inactivating mTORC1 whose activity is influenced by intra-cellular and extra-cellular factors such as iron intake, nutrients, hypoxia, and DNA damage (Knight et al., 2014). Previous studies have shown that inhibition of mTORC1 signaling is a major event that causes defective erythropoiesis and vulnerability to hemolytic crisis. Understanding the molecular mechanisms by which mitochondrial protein synthesis is associated with the mTORC1 activation pathway may provide therapeutic cues for anemia and B-lymphopenia.

In the aspect of clinical relevance to this study, several studies report that mutations and single nucleotide polymorphisms (SNPs) of genes related to mitochondrial DNA, OXPHOS and translation were discovered in human patients exhibiting anemia (Ducamp and Fleming, 2019) and that 78.2% of the patients with mitochondrial disorders had anemia (Finsterer and Frank, 2015), implying a close association between mitochondrial dysfunctions and erythroid differentiation. Although, thus far, mutations or SNPs of p32/C1qbp have found to associate with progressive external ophthalmoplegia or exacerbations of myopathy and influenza infection in human, respectively (Feichtinger et al., 2017) (Chatzopoulou et al., 2018), further studies might reveal genetic anomalies of p32/C1qbp in mitochondrial disorders exhibiting anemia and B-lymphopenia with currently unidentified pathologies.

### Limitations of the Study

In this study, we have shown that mitochondrial p32/C1qbp is essential for terminal differentiation of CD51^–^ TNCs into erythrocytes and B-lymphocytes. In the aspect of mechanisms, we focused on the CD51^–^ TNCs. Genetic deletion of p32/C1qbp in hematopoietic cells causes not only reductions of lymphocytes but also decreases of myeloid cells, which was not significant after the bone marrow transplantation, implying that p32/C1qbp has roles on HSCs. Further investigation under severe hematopoietic stresses as competitive or serial transplantation is required to conclude the functions of p32/C1qbp in HSCs.

### Resource Availability

#### Lead Contact

Further information and requests for resources and reagents should be directed to and will be fulfilled by the Lead Contact, Kazuhito Gotoh (gotou.kazuhito.712@m.kyushu-u.ac.jp).

#### Materials Availability

All mouse lines and reagents generated in this study are available from the Lead Contact with a completed Materials Transfer Agreement.

#### Data and Code Availability

The data that support the findings of this study are available from the Lead Contact upon reasonable requests. RNA sequence data have been deposited to Mendeley Data: https://doi.org/10.17632/w25nchf7cp.1.

## Methods

All methods can be found in the accompanying Transparent Methods supplemental file.

## Supplemental Information

Document S1. Transparent Methods and Figures S1–S7
