# Supplementary material for: Mitochondrial Protein Synthesis Is Essential for Terminal Differentiation of CD45– TER119–Erythroid and Lymphoid Progenitors
Source: iScience. 2020 Oct 7;23(11):101654. doi: 10.1016/j.isci.2020.101654 (PMC7578749; doi:10.1016/j.isci.2020.101654)
Supplement: Document S1. Transparent Methods and Figures S1–S7 [file mmc1.pdf]

iScience, Volume *23*

Supplemental Information

Mitochondrial Protein Synthesis Is Essential for Terminal Differentiation of CD45^{-} TER119^{-} Erythroid and Lymphoid Progenitors

Kazuhito Gotoh, Yuya Kunisaki, Soichi Mizuguchi, Daiki Setoyama, Kentaro Hosokawa, Hisayuki Yao, Yuya Nakashima, Mikako Yagi, Takeshi Uchiumi, Yuichiro Semba, Jumpei Nogami, Koichi Akashi, Fumio Arai, and Dongchon Kang

Figure S1

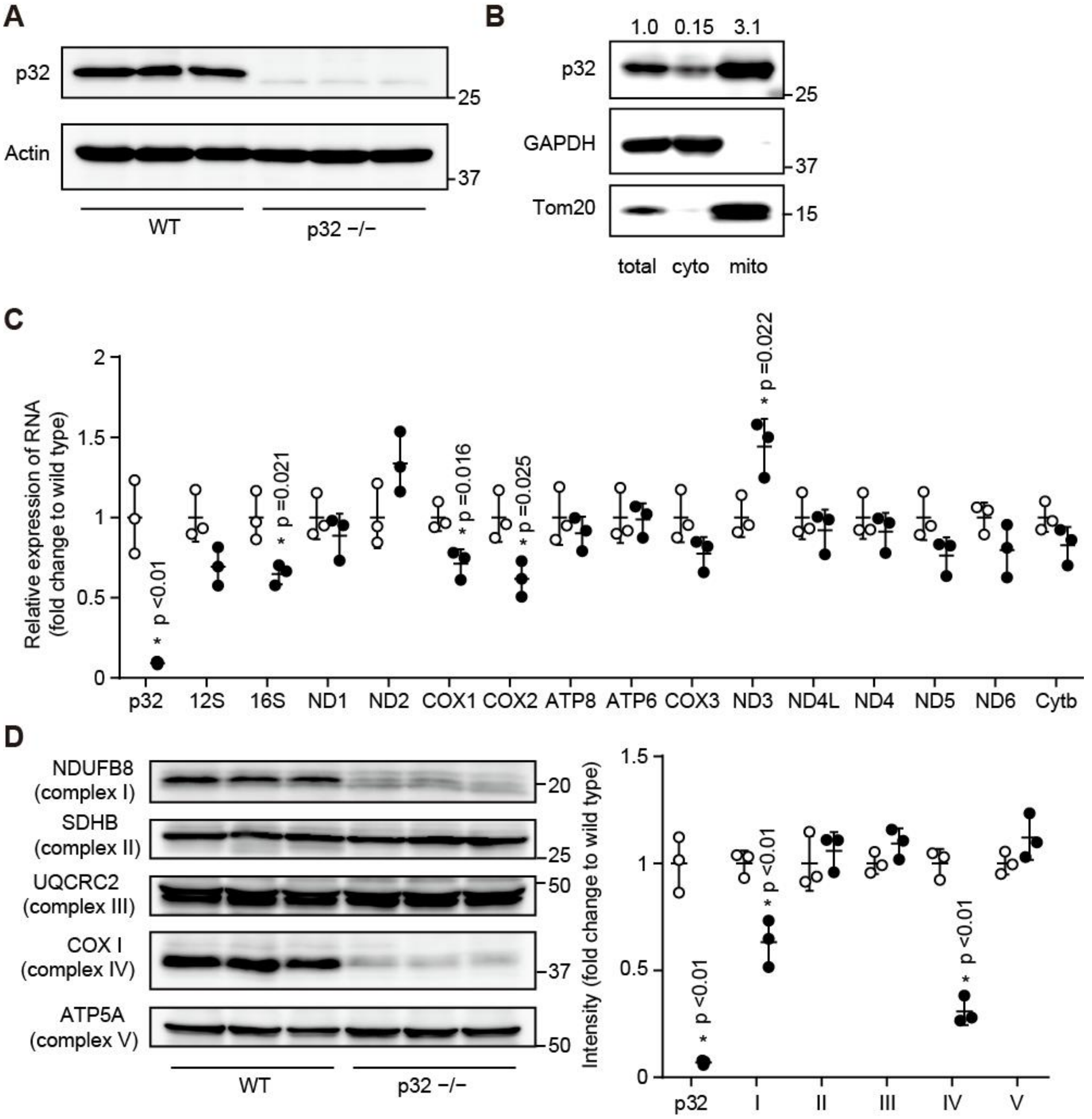
A
p32
Actin
25
37
WT
p32 −/−
B
1.0 0.15 3.1
p32
GAPDH
Tom20
25
37
15
total cyto mito
C
Relative expression of RNA (fold change to wild type)
2
1.5
1
0.5
0
* p <0.01
* p =0.021
* p =0.016
* p =0.025
* p =0.022
p32 12S 16S ND1 ND2 COX1 COX2 ATP8 ATP6 COX3 ND3 ND4L ND4 ND5 ND6 Cytb
D
NDUFB8 (complex I)
SDHB (complex II)
UQCRC2 (complex III)
COX I (complex IV)
ATP5A (complex V)
20
25
50
37
50
WT
p32 −/−
Intensity (fold change to wild type)
1.5
1
0.5
0
* p <0.01
* p <0.01
* p <0.01
p32 I II III IV V

Figure S1. Mitochondrial mRNA, and Protein Expression Levels in BM, Related to Figure 1

(A) Deletion of p32 was analyzed using western blotting in BM from WT and p32cKO mice. β-Actin was evaluated as an internal control.

(B) Subcellular distribution of p32 in BM determined by biochemical fractionation. Tom20 and GAPDH were evaluated as mitochondrial and cytoplasmic markers, respectively.

(C) Real-time PCR quantification of mitochondrial gene transcript levels isolated from BM. Data for each RNA were compared between WT and p32cKO mice after normalization by 18S rRNA expression.

(D) Representative immunoblots ($n = 3$) of mitochondrial respiratory enzyme subunits of complexes I–IV + complex V (H+-ATPase) of BM from WT and p32cKO mice. The mitochondrial respiratory enzyme subunit/β-actin ratios were quantified by normalizing the respiratory enzyme subunit protein levels to the β-actin protein levels.

In (C–D) data are shown as mean ± SD. *$p < 0.05$ versus WT. Data are representative of at least three (A-D) independent experiments.

Figure S2

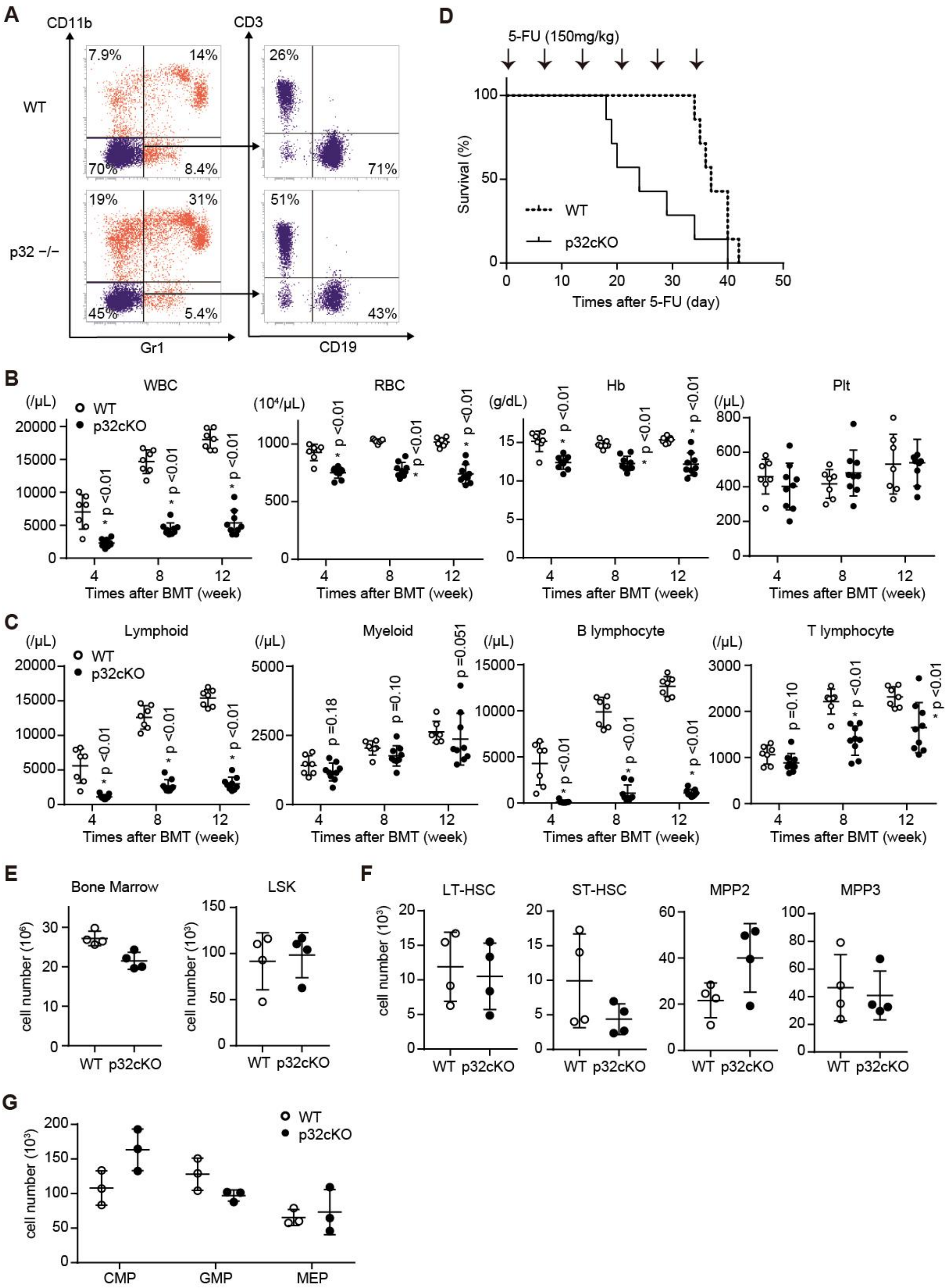

A
CD11b
CD3
WT
p32 −/−
7.9%
14%
70%
8.4%
26%
71%
19%
31%
45%
5.4%
51%
43%
Gr1
CD19
D
5-FU (150mg/kg)
Survival (%)
WT
p32cKO
Times after 5-FU (day)
B
WBC
RBC
Hb
Plt
(/μL)
(10⁴/μL)
(g/dL)
Times after BMT (week)
p <0.01
C
Lymphoid
Myeloid
B lymphocyte
T lymphocyte
p =0.18
p =0.10
p =0.051
E
Bone Marrow
LSK
cell number (10⁶)
cell number (10³)
F
LT-HSC
ST-HSC
MPP2
MPP3
G
CMP
GMP
MEP

Figure S2. FACS analysis of peripheral blood, BM, and HSC, Related to Figure 1 and 2

(A) Gating strategy for the identification of different subpopulations of Myeloid (Gr-1$^+$ CD11b$^+$), B lymphocyte (CD19$^+$ CD3$^-$ Gr-1$^-$ CD11b$^-$) and T lymphocyte (CD19$^-$ CD3$^+$ Gr-1$^-$ CD11b$^-$) in lysed peripheral blood. (B) Bone marrow cells from WT and p32cKO mice were transplanted to lethally irradiated WT mice. WBCs, RBCs, hemoglobin concentration (Hb), hematocrit (Ht), and the platelet (Plt) count in peripheral blood from transplanted mice that received bone marrow cells from WT (open circle, n = 7) and p32cKO (closed squares, n = 9). (C) Numbers of lymphoid cells (Gr-1$^-$ CD11b$^-$), myeloid cells (Gr-1$^+$ CD11b$^+$), B-lymphocytes (CD19$^+$ CD3$^-$ Gr-1$^-$ CD11b$^-$), and T-lymphocytes (CD19$^-$ CD3$^+$ Gr-1$^-$ CD11b$^-$) in the peripheral blood from transplanted mice. (D) Kaplan-Meier plot of Age-matched WT and p32cKO mice (n=7) treated with weekly 5-FU (150 mg/kg). (E-G) The number of BM cells, LSKs (E), LT-HSCs (CD150$^+$ CD48$^-$ Flk2$^-$ LSK), ST-HSCs (CD150$^+$ CD48$^+$ Flk2$^-$ LSK), MPP2s (CD150$^+$ CD48$^-$ Flk2$^-$ LSK), MPP3s (CD150$^-$ CD48$^-$ Flk2$^-$ LSK) (F), CMPs (CD34$^+$ CD16/32$^-$ LK), GMPs (CD34$^+$ CD16/32$^+$ LK), MEPs (CD34$^-$ CD16/32$^-$ LK) (G) in BM from WT and p32cKO mice. Data are representative of at least three (A–G) independent experiments.

Figure S3

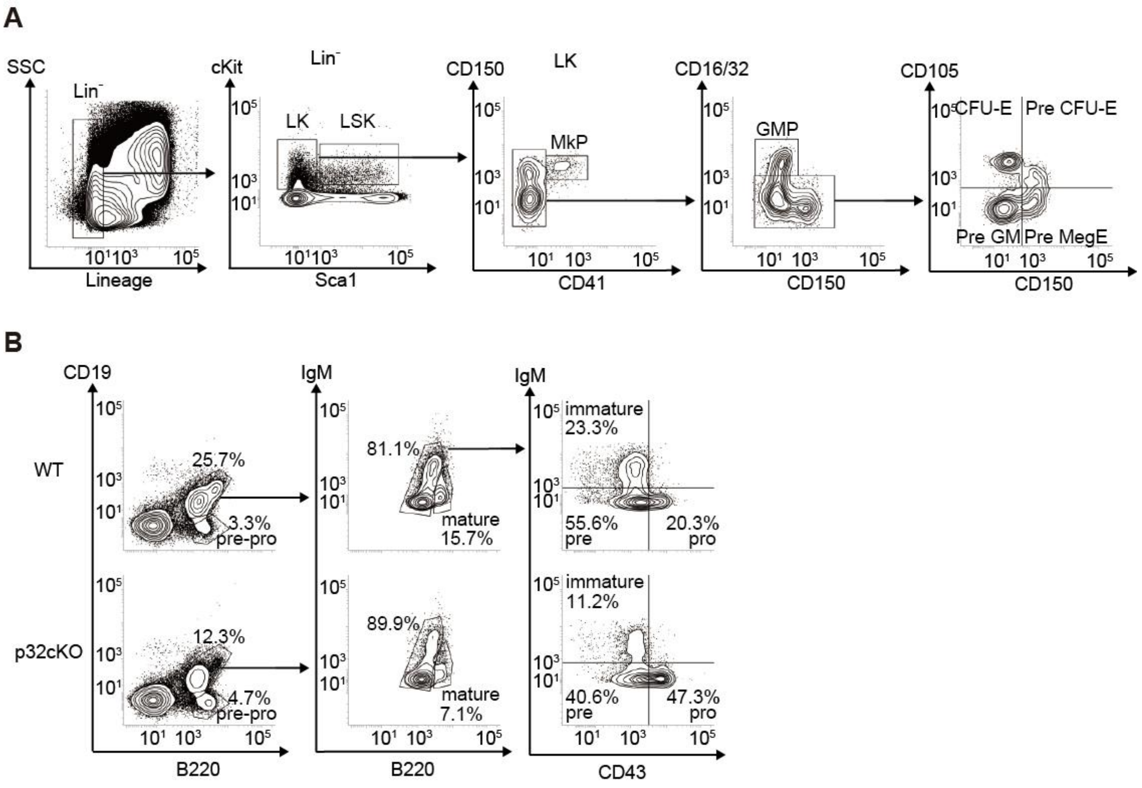

Figure S3. Gating strategies for FACS analysis of Hematopoietic progenitor cells, Related to Figure 2

(A, B) Representative gating strategies for Pre-CFU-E, CFU-E (A), and B lymphoid lineage (B) in BM. Data are representative of <u>three</u> (A, B) independent experiments.

Figure S4

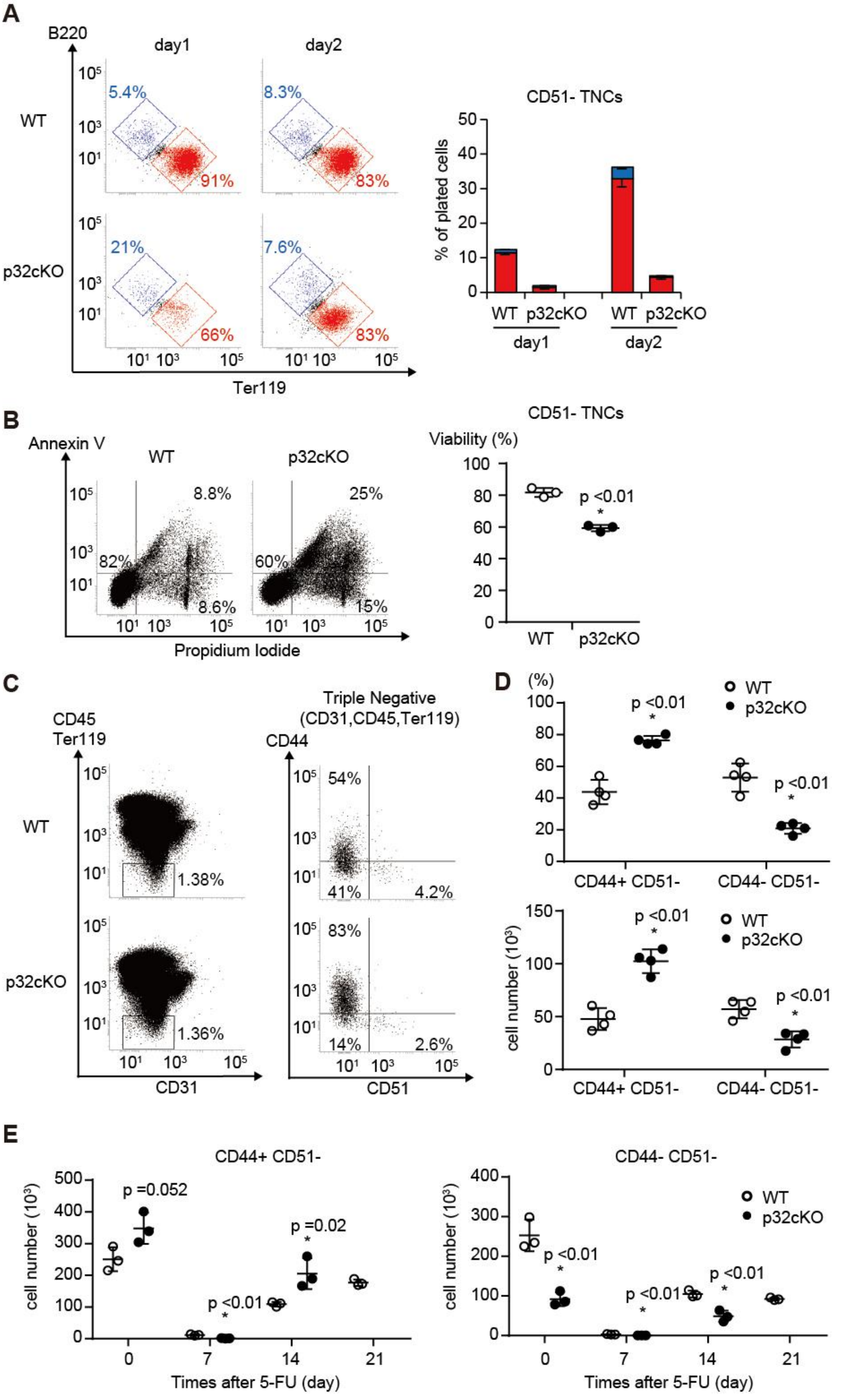

Figure S4. p32/C1qbp is important for CD45^{-} erythroid and B-lymphoid progenitor differentiation, Related to Figure 3

(A) Sorted CD51^{-} TNCs from WT and p32cKO mice were plated in liquid culture with cytokines

(Stem cell factor, IL-3, IL-6, Erythropoietin, Thrombopoietin) for 48 hours. Representative FACS plots (left), proportion (right) of erythroid (Ter119$^+$) and B lymphoid (B220$^+$) lineage potential of sorted CD51$^-$ TNCs. Cell numbers for each population were normalized as a percentage of total cell plated per well (% of cells plated).

(B) FACS analysis of cell death in CD51$^-$ TNCs (left). The rates of the population of Annexin V$^-$/Propidium Iodide$^-$ are indicated (right).

(C, D) Representative flow cytometry plots (C), and the percentages and numbers (D) of CD44$^+$ CD51$^-$ and CD44$^-$ CD51$^-$ TNCs in BM of WT (open circle, n = 3) and p32cKO (closed squares, n = 3) mice. (E) Numbers of CD44$^+$ CD51$^-$ and CD44$^-$ CD51$^-$ TNCs in BM of WT (open circle, n = 3) and p32cKO (closed squares, n = 3) mice after 5-FU injection.

In (A-E) data are shown as mean ± SD. *$p < 0.05$ versus WT mice. Data are representative of three (A-D) independent experiments.

Figure S5

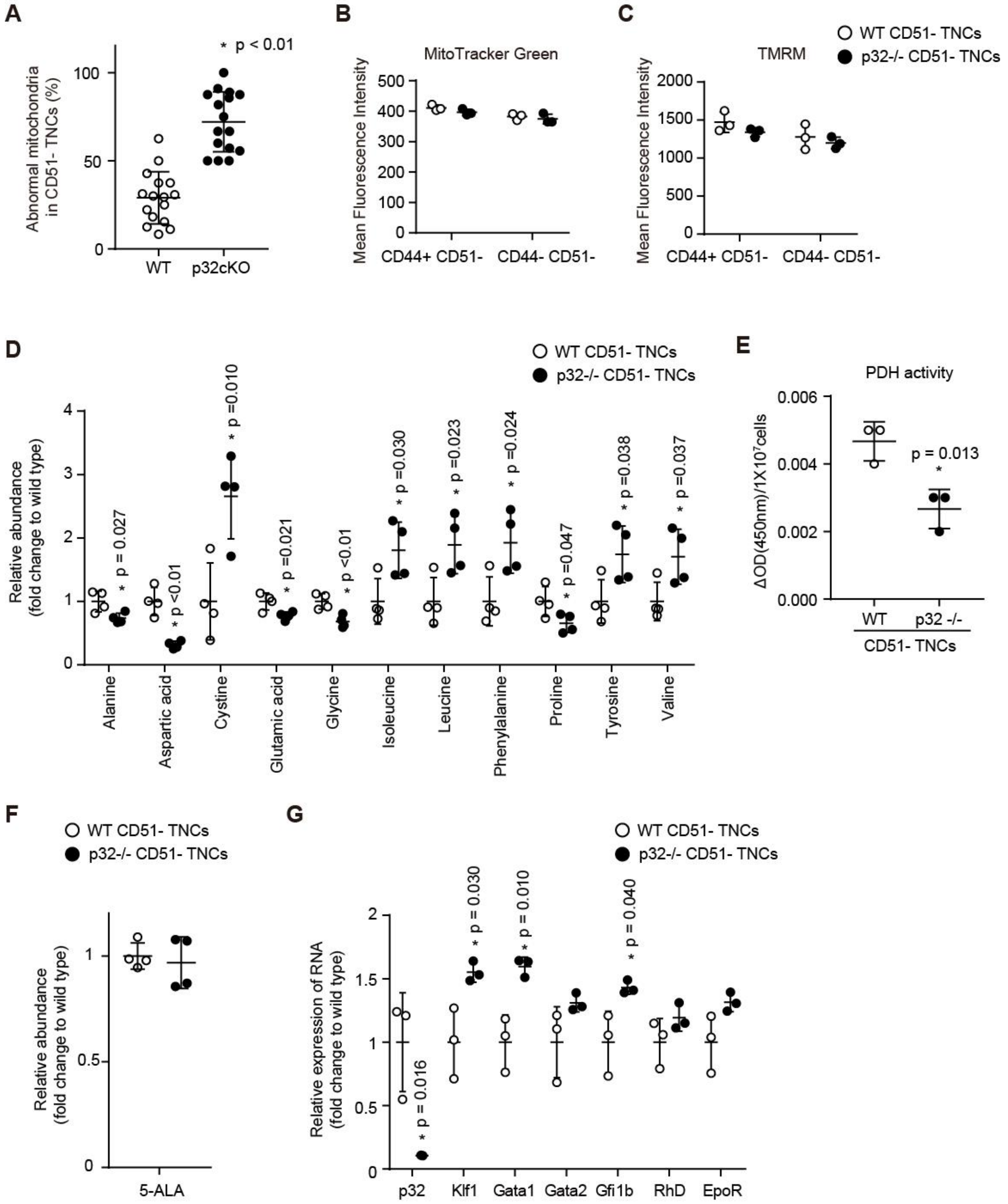

A
* p < 0.01
Abnormal mitochondria in CD51- TNCs (%)
WT
p32cKO
B
MitoTracker Green
Mean Fluorescence Intensity
CD44+ CD51-
CD44- CD51-
C
TMRM
Mean Fluorescence Intensity
CD44+ CD51-
CD44- CD51-
WT CD51- TNCs
p32-/- CD51- TNCs
D
WT CD51- TNCs
p32-/- CD51- TNCs
Relative abundance (fold change to wild type)
* p = 0.027
* p <0.01
* p =0.010
* p =0.021
* p <0.01
* p =0.030
* p =0.023
* p =0.024
* p =0.047
* p =0.038
* p =0.037
Alanine
Aspartic acid
Cystine
Glutamic acid
Glycine
Isoleucine
Leucine
Phenylalanine
Proline
Tyrosine
Valine
E
PDH activity
ΔOD(450nm)/1X10⁷cells
p = 0.013
WT
p32 -/-
CD51- TNCs
F
WT CD51- TNCs
p32-/- CD51- TNCs
Relative abundance (fold change to wild type)
5-ALA
G
WT CD51- TNCs
p32-/- CD51- TNCs
Relative expression of RNA (fold change to wild type)
* p = 0.016
* p = 0.030
* p = 0.010
* p = 0.040
p32
Klf1
Gata1
Gata2
Gfi1b
RhD
EpoR

Figure S5. p32/C1qbp promotes erythroid differentiation of CD51⁻ TNCs by regulating mitochondrial OXPHOS, Related to Figure 4

(A) The ratio of abnormal mitochondria per CD51$^-$ TNCs using electron microscopic images. We calculated abnormal mitochondria in 16 cells per sample.

(B, C) Mitochondrial mass using MitoTracker Green FM (B), Mitochondrial membrane potentials (MMP) using TMRM (C) were measured CD51$^-$ TNCs by FACS analysis. Data are expressed as means ± SD of triplicate samples. *P<0.05, versus WT CD51$^-$ TNCs.

(D, F) Quantification of relative amounts of metabolites in p32$^{-/-}$ CD51$^-$ TNCs compared to WT controls. (E) Enzymatic activities of PDH in WT and p32$^{-/-}$ CD51$^-$ TNCs. (G) qPCR quantification of erythroid gene transcripts isolated from CD51$^-$ TNCs. Data are shown as a relative expression for p32$^{-/-}$ CD51$^-$ TNCs against WT controls. 18S rRNA was used as an internal control.

In (A-G) data are shown as mean ± SD. *p < 0.05 versus WT CD51$^-$ TNCs. Data are representative of at least three (A-G) independent experiments.

Figure S6

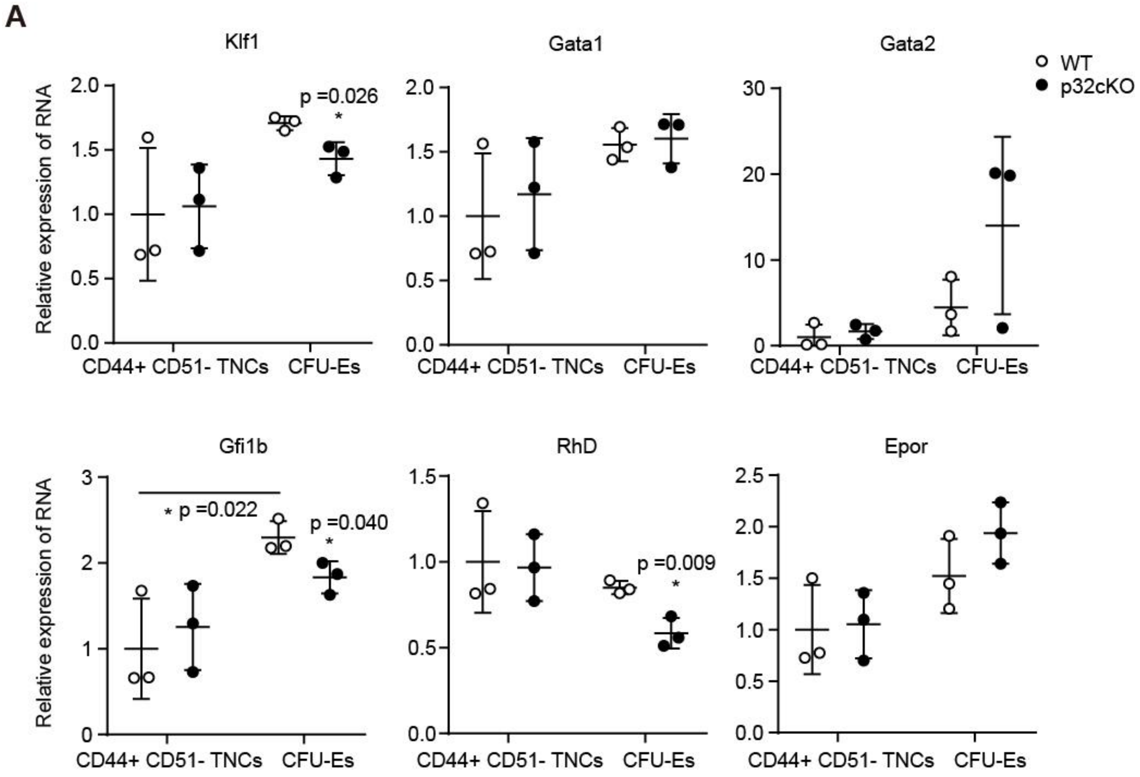
A
Klf1
Gata1
Gata2
WT
p32cKO
Relative expression of RNA
p =0.026
*
CD44+ CD51- TNCs
CFU-Es
Gfi1b
RhD
Epor
* p =0.022
p =0.040
p =0.009

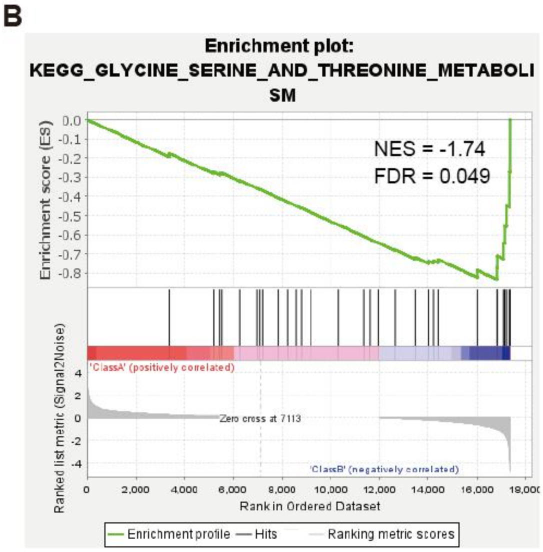
B
Enrichment plot:
KEGG_GLYCINE_SERINE_AND_THREONINE_METABOLISM
NES = -1.74
FDR = 0.049
Enrichment score (ES)
Ranked list metric (Signal2Noise)
Rank in Ordered Dataset
Enrichment profile
Hits
Ranking metric scores

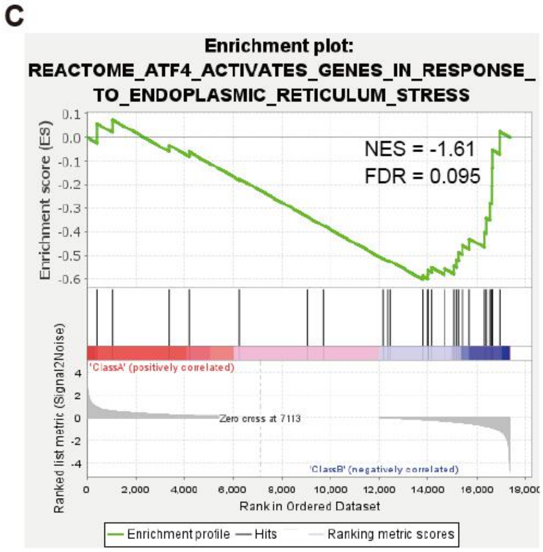
C
Enrichment plot:
REACTOME_ATF4_ACTIVATES_GENES_IN_RESPONSE_TO_ENDOPLASMIC_RETICULUM_STRESS
NES = -1.61
FDR = 0.095
Enrichment score (ES)
Ranked list metric (Signal2Noise)
Rank in Ordered Dataset
Enrichment profile
Hits
Ranking metric scores

Figure S6. Loss of p32/C1qbp promotes mitochondrial stress response, Related to Figure 5 (A) Relative mRNA amount of the genes of erythroid differentiation in $CD44^+$ $CD51^-$ TNCs and CFU-Es isolated from WT (n = 3) and p32cKO (n = 3). Data are shown as a relative expression against WT $CD44^+$ $CD51^-$ TNCs. Data are shown as mean ± SD. *$p < 0.05$ versus WT $CD44^+$ $CD51^-$ TNCs or WT CFU-Es. (B, C) Enrichment score plots from Gene Set Enrichment Analysis (GSEA) of glycine serine and threonine metabolism (B) and ATF4 activates genes in response to endoplasmic reticulum stress (C) of sorted $CD44^+$ $CD51^-$ TNCs from WT (n=3) and p32cKO (n=3) mice. FDR, false discovery rate; Glc, NES, normalized enrichment score. $CD44^+$ $CD51^-$ TNCs and CFU-Es from WT (n = 3) and p32cKO (n = 3) mice were isolated on different days. Further processing and sequencing was performed with all twelve samples simultaneously.

Figure S7

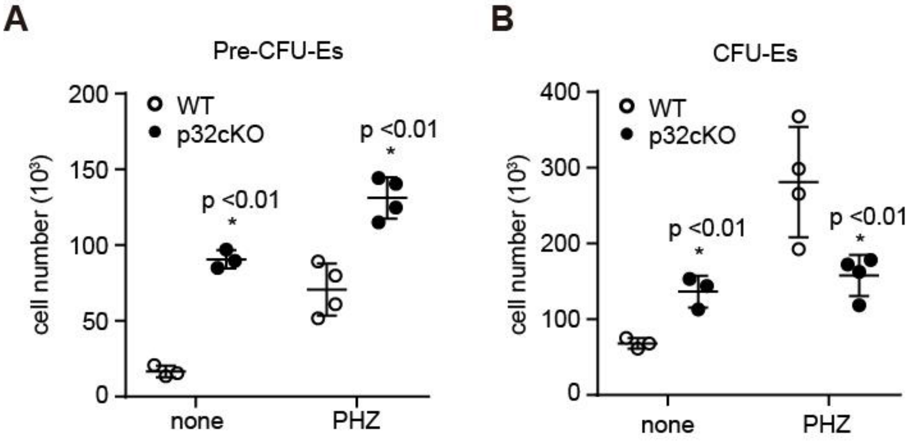

Figure S7. Role of Pre-CFU-Es and CFU-Es after PHZ administration, Related to Figure 7

(A) Numbers of Pre-CFU-Es and CFU-Es in BM of WT (open circle, n = 3) and p32cKO (closed squares, n = 3) mice after PHZ injection. Data are shown as means ± SD. *$p < 0.05$ versus WT mice. Data are representative at least three (A–B) independent experiments.

Transparent Methods

KEY RESOURCES TABLE

REAGENT or RESOURCE	SOURCE	IDENTIFIER
Antibodies		
p32 antibody	Dongchon Kang, Kyushu University	Yagi et al., 2012
β-actin antibody	Merck (Sigma-Aldrich)	Cat# A5441, RRID:AB_476744
Total OXPHOS Rodent WB Antibody Cocktail	abcam	Cat# ab110413, RRID: AB_2629281
COX1 antibody	abcam	Cat# ab14705, RRID:AB_2084810
PE/Cy7 anti-mouse CD3 Antibody	Biolegend	Cat# 100220, RRID: AB_1732057
APC/Cy7 anti-mouse/human CD11b Antibody	Biolegend	Cat# 101226, RRID: AB_830642
PerCP-Cyanine5.5 Anti-Mouse CD19 (1D3) antibody	Tonbo Biosciences	Cat# 65-0193, RRID:AB_2621887
PE/Cy7 anti-mouse Ly-6G/Ly-6C (Gr-1) Antibody	Biolegend	Cat# 108416, RRID: AB_313381
Lineage Cell Detection Cocktail-Biotin, mouse	Miltenyi Biotec	Cat# 130-092-613, RRID:AB_1103214
Anti-Sca-1-APC, mouse	Miltenyi Biotec	Cat# 130-106-425, RRID:AB_2653401
CD117-PE-Vio770	Miltenyi Biotec	Cat# 130-108-355, RRID:AB_2660122
PerCP/Cy5.5 anti-mouse CD41 Antibody	Biolegend	Cat# 133917, RRID:AB_2563499
FITC anti-mouse CD16/32 Antibody	Biolegend	Cat# 101305, RRID:AB_312804
Brilliant Violet 421™ anti-mouse CD150 (SLAM) Antibody	Biolegend	Cat# 115926, RRID:AB_2562190
PE anti-mouse CD105 Antibody	Biolegend	Cat# 120408, RRID: AB_1027699
APC anti-mouse/human CD45R/B220 Antibody	Biolegend	Cat# 103212, RRID:AB_312997
Brilliant Violet 421™ anti-mouse TER-119/Erythroid Cells Antibody	Biolegend	Cat# 116234, RRID:AB_2562917

PE anti-mouse Ly-6D Antibody	Biolegend	Cat# 138604, RRID:AB_2137349
PE/Cy7 anti-mouse CD31 Antibody	Biolegend	Cat# 102418, RRID:AB_830757
APC anti-mouse CD45 Antibody	Biolegend	Cat# 103112, RRID:AB_312977
Biotin anti-mouse CD51 Antibody	Biolegend	Cat# 104104, RRID:AB_313073
APC anti-mouse/human CD44 Antibody	Biolegend	Cat# 103011, RRID:AB_312962
ATF4-human antibody	Cell Signaling Technology	Cat# 11815, RRID:AB_2616025
Sestrin-2 (D1B6) Rabbit mAb antibody	Cell Signaling Technology	Cat# 8487, RRID:AB_11178663
Anti-4E-BP1, phospho (Thr37 / Thr46) Monoclonal Antibody	Cell Signaling Technology	Cat# 2855, RRID:AB_560835
Chemicals, Peptides, and Recombinant Proteins		
5-FU	SIGMA	Cat# F6627
RPMI 1640	SIGMA	Cat# R8758
Penicillin Streptomycin	Thermo Fisher Scientific	Cat# 15140122
L-glutamine	Thermo Fisher Scientific	Cat# 25030081
non-essential amino acids	Thermo Fisher Scientific	Cat# 11140076
sodium pyruvate	Thermo Fisher Scientific	Cat# 11360070
2-mercaptoethanol	Wako	Cat# 137-06862
FCCP	Merck (Sigma-Aldrich)	Cat# C2920
rotenone	Merck (Sigma-Aldrich)	Cat# R8875
antimycin	Merck (Sigma-Aldrich)	Cat# A8674
oligomycin	Merck (Sigma-Aldrich)	Cat# O4876
Torin 1	Selleck Biotech	Cat# S2827
Phenylhydrazine	SIGMA	Cat# P26252
Collagenase IV	GIBCO	Cat# 17104-019
Dispase	GIBCO	Cat# 17105-041
Recombinant murine Scf.	Peprotech	Cat# 250-03
Recombinant murine IL-3	Peprotech	Cat# 213-13
Recombinant murine IL-6	Peprotech	Cat# 216-16
Recombinant murine IL-7	Peprotech	Cat# 217-17
Recombinant murine TPO	Peprotech	Cat# AF-315-14
Recombinant murine EPO	BioLegend	Cat# 587602
Tetramethylrhodamine	Thermo Fisher Scientific	Cat# T668

MitoTracker Red CMXRos	Thermo Fisher Scientific	Cat# M7512
Insulin-Transferrin-Selenium-Ethanolamine	ThermoFisher Scientific	Cat#51500-056
Experimental Models: Organisms/Strains		
p32flox/flox mice	Dongchon Kang, Kyushu University	Yagi et al., 2012
Vav1-iCre mice	Jackson Laboratory	Stock# 8610
C57BL/6 mice	Japan Clea	N/A
Critical Commercial Assays		
MethoCult™ M3630	STEMCELL Technologies	Cat# ST-03630
MethoCult™ M3434	STEMCELL Technologies	Cat# ST-03434
PDH Microplate Assay Kit	abcam	Cat# ab109902
Software and Algorithms		
MetaMorph imaging system	Universal Imaging	N/A
FACSuite software	BD	N/A
GraphPad Prism	GraphPad software	N/A
Gene Set Enrichment Analysis (GSEA)	(Subramanian et al., 2005)	https://www.gsea-msigdb.org/gsea/index.jsp

Contact for Reagent and Resource Sharing

Further information and requests for resources and reagents should be directed to and will be fulfilled by the Lead Contact, Kazuhito Gotoh (gotou.kazuhito.712@m.kyushu-u.ac.jp).

Animals

C57BL/6 mice were purchased from Japan Clea. Vav1-iCre mice (Stock no: 008610) were obtained from Jackson Laboratory. $p32^{flox/flox}$ mice have been described previously (Yagi et al., 2012). Age- and sex-matched $p32^{flox/flox}$ Vav1-iCre^{+} (p32 cKO) and control littermate $p32^{flox/flox}$ Vav1-Cre^{-} (WT) mice were used in this study. All mice were maintained on the C57BL/6 background and kept under specific pathogen-free conditions in the animal facility at Kyushu University. The animal protocols were approved by the Committee of Ethics on Animal Experiments, Faculty of Medical Sciences, Kyushu University.

In vivo treatments

WT and p32cKO mice were matched for age and sex. The mice were anesthetized and intraperitoneally injected with 5-FU (150 or 250 mg/kg body weight) or phenylhydrazine (80 mg/kg body weight) at 8–12 weeks of age. Mouse survival was monitored for up to 60 days after injection. Hematological parameters were determined using a K-4500 automatic analyzer (Sysmex).

For bone marrow transplantation, 1×10^{6} bone marrow cells from WT and p32cKO mice were transplanted into lethally irradiated recipient.

Flow cytometric analysis and cell sorting

To analyze hematopoietic cells, BM cells were flushed and dissociated by gently passing through a 21 G needle. Ammonium chloride was used for red blood cell lysis. To analyze TNCs, BM plugs were flushed and digested sequentially in HBSS buffer containing collagenase type IV (2 mg/mL, GIBCO) and dispase (1 mg/mL, GIBCO) three times for 10 minutes each at 37°C. The supernatant was collected between digestions and pooled in a tube containing ice-cold FACS buffer (PBS with EDTA 2 mM, BSA 0.1%, and 0.05% NaN_3). Cells were stained in PEB buffer (PBS with 0.5% BSA and 2 mM EDTA) for 30 min on ice. Multiparametric flow cytometric analyses were performed on a FACS Verse with BD FACSuite software (BD Biosciences). Data were analyzed by FlowJo software (Tree Star). Cell sorting was performed using an SH800 (Sony) and Aria Cell Sorter (BD).

For intracellular staining, BM cells were fixed with 4% (wt/vol) paraformaldehyde/PBS (Wako Pure Chemical Industries) and permeabilized with 0.2% (wt/vol) Triton X-100/PBS for 15 min at room temperature. After blocking with 1% bovine serum albumin (BSA)/PBS for 30 min, the cells were incubated with primary antibodies in 1% BSA/PBS for 1 hour. Then, the cells were washed with PBS and incubated with an Alexa 488-labeled anti-rabbit secondary antibody for 1 hour.

Immunoblot analysis

For direct immunoblotting, BM cells were lysed in cell lysis buffer (Cell Signaling Technology) and subjected to immunoblotting using specific antibodies.

Quantitative real-time PCR analyses

Total RNA was extracted with an RNeasy Tissue Kit (QIAGEN) and CellAmp Direct Lysis and RT Kit (Takara). Reverse transcription of approximately 650 ng total RNA was performed with random hexamer primers using a PrimeScript RT Reagent Kit (Takara). Expression of mitochondrial genes was detected by qPCR with a thermal cycler (StepOne plus; Applied Biosystems). Ribosomal 18S rRNA was evaluated as an internal control.

Cell culture assays

CFU-E and CFU-pre-B cells, $CD51^-$ TNCs, and $Ly6D^-$ $CD44^+$ $CD51^-$ TNCs (1000 cells per plate) were individually sorted from BM of WT and p32cKO mice. Sorted cells were plated in methylcellulose [Stem Cell Technologies, Cat#: 3630 (CFU-pre-B cells)] with stem cell factor (25 ng/mL, Cat#: 3434, CFU-E cells) and incubated for 5–7 days (CFU-pre-B cells) or 2 days (CFU-E cells) at 37°C in 5% CO_2.

Sorted CFU-E cells, pre-CFU-E cells, $CD51^-$ TNCs, and $Ly6D^-$ $CD44^+$ $CD51^-$ TNCs from WT and p32cKO mice (1000 cells/mL) were cultured in 24-well plates containing DMEM (Sigma) supplemented with 30% FBS (Sigma), 100 U/mL penicillin-streptomycin (Nacalai Tesque), 1-thioglycerol 0.1 mM (Sigma), insulin-transferrin-selenium-ethanolamine (ITS-X, Thermo Fisher Scientific), 2 U/mL erythropoietin (BioLegend), 25 ng/mL stem cell factor (PeproTech), 25 ng/mL IL-3 (PeproTech), 25 ng/mL IL-6 (PeproTech), 25 ng/mL thrombopoietin (PeproTech), and 25 ng/mL IL-7 (PeproTech) under normoxia. The lineage potential of sorted TNCs and erythroid progenitors was measured by flow cytometry using B-lymphoid ($B220^+$) and erythroid ($Ter119^+$) markers. Cell numbers in each population were normalized as the percentage of total cells plated per well (% of cells plated).

Transmission electron microscopy

Sorted $CD51^-$ TNCs were immersed in 0.1 M cacodylate buffer containing 2.5% glutaraldehyde at room temperature overnight. Samples were post-fixed in 0.1 M sucrose buffer containing 1% OsO_4 at 4°C for 2 hours. Tissue samples were dehydrated in a graded ethanol series. Ultrathin sections were prepared with an ultramicrotome (EM UC7, Leica) and stained with 2% uranyl acetate and lead citrate. The sections were visualized under a transmission electron microscope (Tecnai 20, FEI Co.).

Metabolism assays

Sorted $CD51^-$ TNCs were analyzed using an XF-24 Extracellular Flux Analyzer (Seahorse Bioscience). Briefly, $CD51^-$ TNCs were seeded in poly-L-lysine-coated XF-24 well culture plates (200,000 cells/well). At the specified time points, sorted $CD51^-$ TNCs were washed and analyzed in XF Running Buffer (unbuffered RPMI medium with 10 mM glucose, 10% fetal calf serum, and 2 mM L-glutamine) according to the manufacturer's instructions to obtain real-time measurements of the OCR and ECAR. Analyses of the ECAR and/or OCR in response to 0.25 μM oligomycin, 10 μM FCCP, and 1 μM rotenone plus 1 μM antimycin A were performed.

For PDH activities, total cell lysates from 1×10^7 $CD51^-$ TNCs/sample were subjected to microplate assays (PDH Microplate Assay Kit, ab109902; Abcam). The activities were determined by the conversion rates of NAD+ to NADH through coupling with a reporter dye, and the absorbance changes were recorded at 450 nm (SPECTROstar Nano; BMG LabTech).

Metabolite extraction from $CD51^-$ TNCs.

$CD51^-$ TNCs ($8\text{-}10\times10^5$ cells) were prepared from bone marrows of 5-10 mice in each group (WT and p32cKO), respectively, sorted, washed twice with PBS, and frozen in liquid nitrogen. Water-soluble metabolites were extracted from these cell pellets in 300 μL of ice-cold 90% methanol with three times sonication (30 sec of sonication and 30 sec cooling) using a BIORUPTOR (Cosmo Bio Co., Ltd, Japan) and centrifugation at 21,500 g for 5 min at 4 °C. The supernatants were then evaporated to dryness on a miVac DUO concentrator (GeneVac). Desiccated pellets were dissolved in 50 μL of 0.1% formic acid and 10 μL (equivalent to 2×10^5 cells) was used for LC-MS analysis.

LC-MS analysis.

The samples were analyzed by LC-MS based on reverse phase chromatography coupled with a triple quadrupole mass spectrometer LCMS-8060 (Shimadzu, Japan). A reverse phase chromatography was performed using a Discovery HS-F5-3 column (150×2.1 mm, 3 μm particle size, Sigma-Aldrich) with mobile phases consisting of solvent A (0.1% formic acid) and solvent B (0.1% formic acid in acetonitrile). The column oven temperature was 40 C. The gradient elution program was as follows: a flow rate of 0.25 mL/min: 0-2min, 0%B; 2-5min, 0-25%B; 5-11min, 25-35%B; 11-15min, 35-95%B; 15-25min, 95%B; 25.1-30min, 0%B. The parameters for the heated electrospray ionization source in negative/positive ion mode under multiple reaction monitoring (MRM) were as follows; drying gas flow rate, 10 L/min; nebulizer gas flow rate, 3 L/min; heating gas flow rate, 10 L/min; interface temperature, 300 C; DL temperature, 250 C; and heat block temperature, 400 C; CID gas, 270kPa. Data processing was carried out using

LabSolutions LC-MS software program (Shimadzu, Japan).

RNA preparation and next-generation sequencing

Total RNA from sorted 1×10^4 $CD44^+$ $CD51^-$ TNCs and 5×10^3 CFU-Es was extracted using the NucleoSpin RNA Plus XS kit (Machery Nagel). Complementary DNA was generated using the SMART-Seq v4 Ultra Low Input RNA Kit for Sequencing (Clontech Laboratories) from totalRNA. The Nextera XT DNA Sample preparation Kit (Illumina) was used for preparation of DNA libraries. For each sample, the library was sequenced with an Illumina next-generation sequencing system NextSeq 500. The reads were mapped against the mouse reference genome (mm10) by HISAT2 (v. 2.0.4) (Kim et al., 2015), and the expression levels of all genes were quantified by using featureCounts (v. 1.6.3) (Liao et al., 2014). The differential expression analysis was conducted by DESeq2 (v. 1.22.2) (Love et al., 2014).

Supplemental References

Kim, D., Langmead, B., and Salzberg, S.L. (2015). HISAT: a fast spliced aligner with low memory requirements. Nature methods *12*, 357-360.

Liao, Y., Smyth, G.K., and Shi, W. (2014). featureCounts: an efficient general purpose program for assigning sequence reads to genomic features. Bioinformatics *30*, 923-930.

Love, M.I., Huber, W., and Anders, S. (2014). Moderated estimation of fold change and dispersion for RNA-seq data with DESeq2. Genome Biol *15*, 550.

Subramanian, A., Tamayo, P., Mootha, V.K., Mukherjee, S., Ebert, B.L., Gillette, M.A., Paulovich, A., Pomeroy, S.L., Golub, T.R., Lander, E.S., et al. (2005). Gene set enrichment analysis: a knowledge-based approach for interpreting genome-wide expression profiles. Proc. Natl. Acad. Sci. U. S. A. 102, 15545-15550.

Yagi, M., Uchiumi, T., Takazaki, S., Okuno, B., Nomura, M., Yoshida, S., Kanki, T., and Kang, D. (2012). p32/gC1qR is indispensable for fetal development and mitochondrial translation: importance of its RNA-binding ability. Nucleic acids research *40*, 9717-9737.
